# From Artificial to Embodied and Physical Intelligence in Smart Manufacturing

**DOI:** 10.1002/advs.78384

**Published:** 2026-10-26

**Authors:** Kang Wang, Xinyu Lu, Tong Liu, Jinjie Fang, Xin Li, Siqian Wu, Liuchao Jin, Wei‐Hsin Liao, Shuyou Zhang, Jianrong Tan

**Affiliations:** ^1^ State Key Laboratory of Fluid Power and Mechatronic Systems School of Mechanical Engineering Zhejiang University Hangzhou Zhejiang China; ^2^ Zhejiang Key Laboratory of Advanced Equipment Manufacturing and Measurement Technology School of Mechanical Engineering Zhejiang University Hangzhou Zhejiang China; ^3^ School of Aeronautics and Astronautics Zhejiang University Hangzhou Zhejiang China; ^4^ Department of Mechanical and Automation Engineering The Chinese University of Hong Kong Hong Kong China; ^5^ Institute of Intelligent Design and Manufacturing The Chinese University of Hong Kong Hong Kong China

**Keywords:** smart manufacturing, artificial intelligence, embodied intelligence, physical intelligence, machine learning, smart materials, digital twins

## Abstract

Manufacturing is evolving from automation‐centered production toward systems capable of sensing, adaptation, and autonomous response across digital, mechanical, and material domains. Although recent progress has been largely driven by artificial intelligence, current manufacturing intelligence often remains external to physical systems and suffers from limited robustness, interpretability, and transferability in complex industrial environments. Here, we present a unified perspective on smart manufacturing through the framework of artificial intelligence (AI), embodied intelligence (EI), and physical intelligence (PI). AI provides abstraction, prediction, and generative capability; EI enables closed‐loop perception, decision‐making, and physical interaction; and PI embeds adaptive intelligence directly into materials, structures, and physicochemical processes. We further discuss how generative intelligence is beginning to connect these domains through inverse design, adaptive fabrication, and physics‐constrained behavior realization. Recent advances in collaborative robotics, embodied digital twins, self‐optimizing production systems, and physically intelligent materials, including self‐actuating, self‐healing, multistable, and shape‐morphing systems, are reviewed. Finally, we propose an integrated AI–EI–PI framework spanning the full manufacturing lifecycle, highlighting a transition toward manufacturing systems in which intelligence is increasingly co‐designed across algorithms, embodied agents, and matter itself.

## Graphical abstract

Smart manufacturing is reframed through complementary artificial, embodied, and physical intelligence, spanning computational reasoning, perception‐action coupling, and material‐level adaptation. Their integration across design, fabrication, operation, and maintenance reveals how intelligence can be co‐designed across algorithms, embodied agents, and matter, while highlighting the interface bottlenecks that govern progress toward adaptive, resilient manufacturing ecosystems.

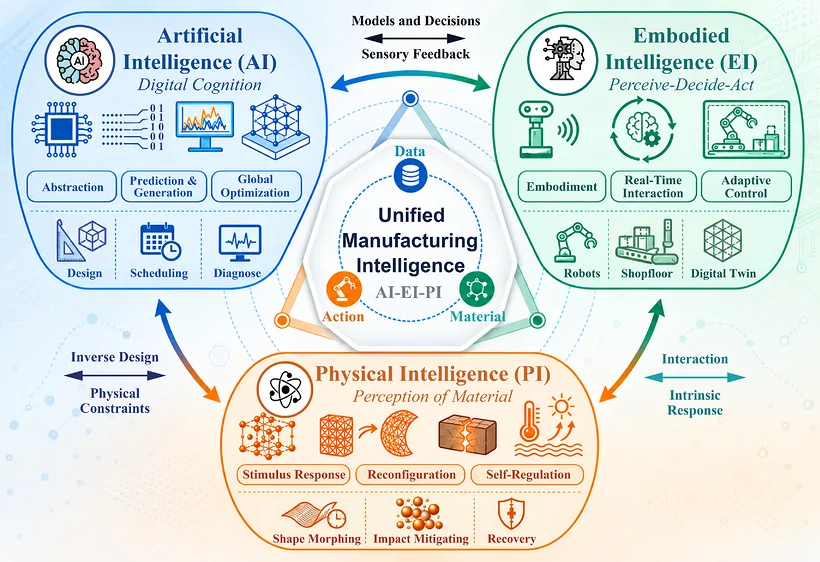

## 1 Introduction

Smart manufacturing is undergoing a fundamental transition, distributing intelligence across algorithms, machines, and materials. Systems once built around automation are being reconfigured into adaptive, connected and increasingly autonomous forms of production [1]. Over the past decade, advances in cyber–physical systems [2, 3, 4, 5, 6, 7], the industrial internet of things [8, 9, 10, 11, 12], artificial intelligence [13, 14, 15], and digital twins [16, 17, 18, 19, 20, 21, 22] have made it possible for manufacturing platforms to sense, model and respond to processes with far greater fidelity and speed. This technological base has helped drive the rise of Industry 5.0 [23, 24, 25, 26, 27, 28], which extends the goals of industrial intelligence beyond productivity and automation alone. In this view, future manufacturing systems are expected to be not only efficient, but also human‐centered, resilient, and sustainable. Therefore, intelligence in manufacturing is no longer a mere matter of computation, for it now spans data interpretation, physical interaction and intrinsic material response.

Despite this shift, most current approaches to smart manufacturing still place artificial intelligence (AI) at the center [29, 30, 31, 32]. In many cases, intelligence is defined by the ability to collect and process data, train models, and use those models for perception, prediction, optimization, and decision‐making. This strategy has led to substantial progress in generative design [33, 34], process monitoring [35, 36, 37], defect detection [38, 39], predictive maintenance [40, 41, 42], and production scheduling [43, 44, 45, 46]. But the intelligence produced in this way often remains external to the physical system itself. Models are typically trained on representations of manufacturing processes rather than on the physical mechanisms and self‐responsive behaviors that govern them. As a result, many AI‐driven manufacturing systems still depend heavily on large, carefully curated datasets, remain sensitive to shifts in operating conditions, and struggle to generalize across materials, scales, machines, and environments. These limitations become especially visible in industrial settings, where data are often scarce, labels are costly, and disturbances are difficult to control. This has led to a growing recognition that the next stage of smart manufacturing will require forms of intelligence that are more physically grounded, more interpretable, and more adaptive than traditional closed‐loop sensing and actuation paradigms.

This need has brought increasing attention to embodied intelligence [47, 48, 49, 50]. At its core, embodied intelligence (EI) treats intelligent behavior as the emergence from continuous coupling among sensing, action, morphology, and the environment, rather than from computation alone [51]. Manufacturing systems must also act, adapt, and collaborate in dynamic, uncertain physical settings using robots, controllers, sensors, actuators, and networked infrastructures [52]. In such systems, intelligent behavior is often inseparable from the physical entities through which decisions are carried out. From this perspective, intelligence is not simply computed in a central model and then issued as a command, but is realized through a perception–decision–action loop in which the body, the controller, and the environment jointly shape system behavior [53]. Embodied intelligence, therefore, provides a crucial bridge between abstract reasoning and real‐world manufacturing execution.

Even this view often treats the physical body primarily as a carrier of sensing and actuation rather than as a source of intelligence in its own right. This motivates a further step toward physical intelligence (PI), in which intelligence is not only deployed onto matter but built into it [54, 55, 56]. In PI‐based systems, sensing, information processing, actuation, and adaptation can be achieved, at least in part, and in some cases predominantly, through the intrinsic physicochemical and structural properties of materials and architectures themselves [57, 58, 59]. Such capabilities are increasingly visible in 4D‐printed systems [60, 61, 62, 63, 64], smart materials [65, 66, 67], self‐healing matter [68, 69, 70, 71, 72], shape‐morphing structures such as origami‐ and kirigami‐inspired architectures [73, 74, 75], and multistable mechanical systems [76, 77]. These classes should be understood as enabling physical platforms rather than PI functions by themselves. In the functional framing adopted in this Review, PI is expressed through task‐relevant capabilities such as stimulus response, physical reconfiguration, and self‐regulation, which can produce outcomes including shape morphing, impact mitigation, and functional recovery. In these systems, function is not encoded only in control software. It is also encoded in geometry, composition, interfaces, and manufacturing history. The boundaries between sensor, controller, computational unit, and actuator, therefore, begin to blur: deformation can serve as sensing, response as computation, and structure as memory or logic, but only when those functions are physically instantiated rather than computed in an external model. In manufacturing systems, physical intelligence should be understood not only as intelligence embedded into matter, but as intelligence continuously generated and reorganized through the interaction between materials, processes, machines, and environments across the entire lifecycle.

This transition from algorithm‐centered intelligence to embodied, physically embedded intelligence is highly relevant to smart manufacturing, but the literature remains fragmented. AI in manufacturing is commonly discussed in terms of machine learning, optimization, and digital workflows [78, 79, 80, 81]. Embodied intelligence is often framed through robotics, human–machine interaction, and cyber–physical systems [82, 83, 84, 85]. Physical intelligence, by contrast, is usually developed within materials science, soft matter, mechanical metamaterials, and responsive structures [86, 87, 88]. Although these areas are advancing rapidly, they are rarely brought together within a common framework that explains how intelligence can be distributed across computational models, embodied systems, and physical matter over the full manufacturing lifecycle. As a result, current discussions often miss the deeper continuity between data‐driven cognition, embodied execution, and material‐level adaptation, even though these capabilities are increasingly converging in next‐generation manufacturing platforms.

In this Review, we present a unified perspective on smart manufacturing that brings together artificial intelligence (AI), embodied intelligence (EI), and physical intelligence (PI) (Figure 1). We argue that future manufacturing systems will be defined not by any one of these forms of intelligence in isolation, but by their coordinated co‐evolution. We first clarify the conceptual foundations of AI, EI, and PI in manufacturing, with emphasis on their distinct modes of information processing, interaction, and adaptation. We then examine how generative intelligence is beginning to connect digital cognition with physically executable behavior, linking inverse design, process planning, and adaptive fabrication. Next, we review recent progress in embodied intelligence for manufacturing systems and in physical intelligence across the manufacturing lifecycle, focusing on how intelligence can be distributed across machines, structures, materials, and processes. Finally, we synthesize these developments into a unified AI–EI–PI framework and outline future opportunities for self‐evolving, material‐aware, and physically embedded manufacturing ecosystems. By treating manufacturing intelligence as complementary computational, embodied, and material implementations, this Review aims to provide a conceptual foundation for the next generation of manufacturing systems that are adaptive by design, interactive by embodiment, and intelligent by matter.

**FIGURE 1:**
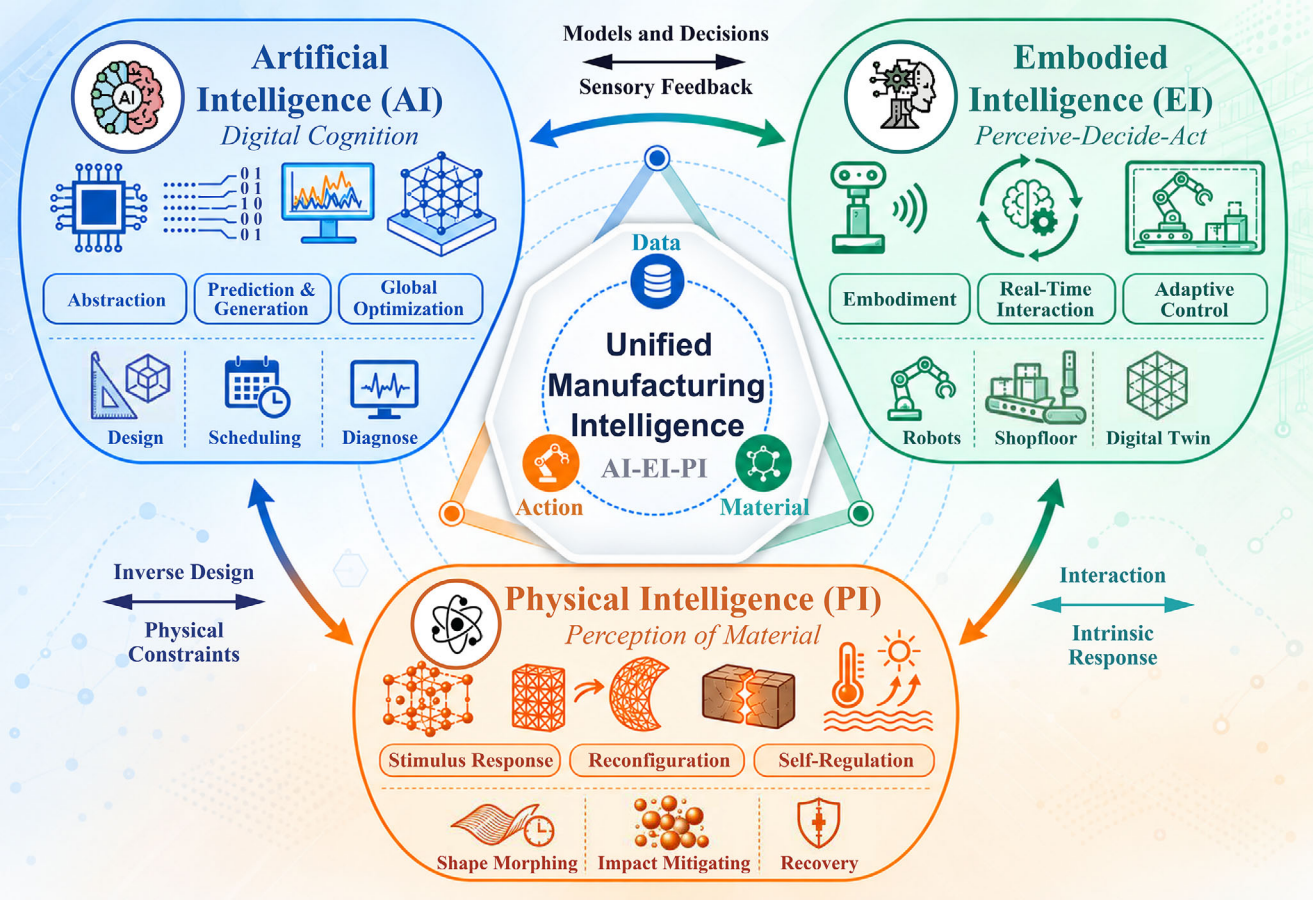
Conceptual organization and coupling of AI, EI, and PI in smart manufacturing. AI (left) provides digital cognition through abstraction, prediction and generation, and global optimization, supporting applications such as design, scheduling, and diagnostics. EI (right) realizes perception–decision–action loops through embodiment, real‐time interaction, and adaptive control in robots, shop‐floor systems, and digital twins. PI (bottom) realizes physically embedded functionality through stimulus response, physical reconfiguration, and self‐regulation, supporting task‐relevant outcomes such as shape morphing, impact mitigation, and functional recovery.

## 2 Evolution of AI, EI, and PI

The progression of intelligence within complex systems is examined here across computational cognition, embodied interaction, and physical realization. This progression represents an accumulation of complementary capabilities rather than the replacement of one form of intelligence by another. As defined in the overarching framework, this evolution is categorized into three analytically distinct but interacting stages: Stage 1 is AI‐driven (data‐centric intelligence), which focuses on high‐dimensional pattern recognition and computational cognition; Stage 2 is EI‐driven (interaction‐centric intelligence), which enables real‐time perception–action coupling through physical morphology; and Stage 3 is PI‐driven (physics‐embedded intelligence), which distributes selected functional responses within the physicochemical substrate of the system [89].

The driving force behind this evolution resides in the escalating requirement for system autonomy, response fidelity, and operational resilience across diverse spatial and temporal scales. Artificial Intelligence, as the starting point, provides the capacity for global optimization and abstract reasoning within virtual manifolds [13]. However, when AI models are insufficiently grounded in physical processes, purely data‐driven models are vulnerable to unmodeled environmental stochasticity and distribution shifts. This limitation motivates closer integration with Embodied Intelligence, which extends cognitive capabilities into the physical domain through sensory‐motor loops [51, 90]. Yet, even within the EI paradigm, many adaptive functions remain mediated by discrete sensing, computation, and actuation, with associated communication, calibration, and energy costs. Consequently, the further evolution toward Physical Intelligence is driven by the need for rapid and distributed local responses under specific operating conditions, where the material substrate itself participates in the mapping from stimulus to functional response [91].

Figure 1 organizes AI, EI, and PI according to their complementary roles in smart manufacturing. AI supports computational abstraction, prediction and generation, and global optimization; EI realizes perception–decision–action loops through embodiment, real‐time interaction, and adaptive control; and PI realizes physically embedded functionality through stimulus response, physical reconfiguration, and self‐regulation, enabling task‐relevant outcomes such as shape morphing, impact mitigation, and functional recovery. The central framework integrates these domains through data, action, and material processes. The bidirectional interfaces represent models and sensory feedback between AI and EI, inverse design and physical constraints between AI and PI, and physical interaction and intrinsic response between EI and PI.

### 2.1 Artificial Intelligence (AI): The Cognitive Starting Point

Artificial Intelligence (AI) serves as the foundational cognitive infrastructure and the initial starting point within the evolutionary continuum of intelligent systems [92, 93]. Operating primarily as a computational layer implemented on digital hardware, AI has progressively evolved from isolated analytical algorithms and deterministic rule‐based expert systems into a generalized computational framework. This modern framework is characterized by highly overparameterized deep neural networks, reinforcement learning policies, and generative foundation models [33, 94]. In this stage, intelligence is instantiated primarily through computational models and digital hardware that process heterogeneous multi‐modal data, encompassing temporal frequency signals, topological structures, optical pixel arrays, and discrete semantic text. By employing complex mathematical optimization architectures—such as gradient‐based backpropagation traversing highly non‐convex loss landscapes, and multi‐head self‐attention mechanisms—AI derives operational intelligence from data, encoded knowledge, and, increasingly, physical constraints. This computational paradigm enables systems to extract complex latent correlations, perform high‐dimensional topological dimensionality reduction, and construct predictive, generative, and decision‐oriented models [95].

From an information‐processing perspective, AI fundamentally operates within symbolic and data‐centric spaces. The information flow intrinsic to AI is defined by a rigorous requirement for abstraction, symbolization, and mathematical discreteness [96]. Continuous physical phenomena, thermodynamic fluctuations, and environmental dynamics must first be rigorously converted into digital representations—encoded as discrete binary bits—through specialized sensing and data acquisition pipelines before any algorithmic reasoning can occur. This structural transformation effectively allows AI systems to process “digital representations of the physical world” rather than directly interact with the continuous physical world [97]. The primary computational advantage of this decoupling resides in its abstraction capability. By mapping physical states onto lower‐dimensional latent manifolds, AI enables the efficient exploration of vast solution spaces and the execution of global optimization strategies across complex probabilistic networks without the temporal and material costs of physical trial‐and‐error. Within these strictly virtual spaces, AI can autonomously synthesize structural topologies, optimize temporal scheduling, and formulate operational policies, effectively transforming raw numerical observations into actionable digital knowledge [98].

Despite these robust cognitive computational capabilities, the purely data‐centric paradigm possesses important deployment limitations when deployed in non‐stationary, dynamic, and uncertain physical environments. The most prominent constraint is strong data dependency. Contemporary AI architectures often require large‐scale, high‐quality, and carefully curated labeled datasets to establish reliable predictions [99, 100, 101, 102]. In complex physical systems, critical boundary cases, transient mechanical anomalies, or system failures occur infrequently, resulting in highly imbalanced datasets and sparse experimental observations. Consequently, many AI models remain strongly dependent on the range of their training data. They can exhibit limited generalization or increased predictive uncertainty when subjected to out‐of‐distribution shifts caused by unmodeled environmental fluctuations, sensor noise drift, or variable physical boundary conditions. This statistical reliance dictates that the intelligence generated is influenced by historical states and may not extrapolate reliably into unseen operational domains.

Furthermore, a more profound challenge is limited intrinsic physical grounding. Because AI operates predominantly in symbolic latent spaces, it typically externalizes intelligence from the physical substrate. The algorithms excel at identifying abstract statistical correlations but do not automatically enforce physical causality, thermodynamic constraints, solid mechanics, or multi‐physics coupling phenomena. Standard neural networks do not natively incorporate the governing differential equations—such as Navier–Stokes equations for fluid dynamics or Hamiltonian mechanics for energy conservation—unless they are explicitly introduced through model architecture, training objectives, or hybrid modeling [103, 104]. This structural separation between computational reasoning and physical reality creates a persistent and well‐documented “sim‐to‐real” discrepancy. Mathematical solutions that achieve strong performance in latent simulation environments may be unstable, mechanically infeasible, or operationally unreliable when subjected to actual rigid‐body dynamics and material friction [105].

Because AI cannot by itself sense contact or act on a workpiece, a critical execution gap remains open at this stage. AI systems often function as upstream computational systems that output abstract behavioral policies, symbolic digital intents, and macro‐level design strategies [106]. These computationally generated outputs must be translated into physical mechanical motion or material phase alteration. Coupling these computational outputs with sensing, control, and physical execution provides the bridge toward Embodied Intelligence [51, 104].

From an application perspective, inspection, condition monitoring, and scheduling represent comparatively mature uses of AI in manufacturing, whereas generative process planning, cross‐machine transfer, and autonomous closed‐loop optimization remain more dependent on data coverage, uncertainty estimation, and verification of physical feasibility.

### 2.2 Embodied Intelligence (EI): Extending Cognition to Physical Interaction

Embodied Intelligence (EI) emerges as a physically situated extension of artificial intelligence, directly addressing the execution gap and the physical decoupling inherent in purely data‐centric computational models [47]. While AI resides primarily within abstract, high‐dimensional representation spaces, EI instantiates cognition within a defined physical morphology, fundamentally shifting the system's operational paradigm from isolated computational inference to interaction‐centric physical execution. By embedding computational logic into physically interactive agents—such as multi‐axis robotic manipulators, autonomous mobile platforms, and adaptive execution units—EI establishes a continuous “Perception–Decision–Action” loop [47, 107]. This closed‐loop architecture serves as the critical mediating layer that translates the macro‐level intents and symbolic policies generated by upstream AI models into executable, physically grounded dynamic behaviors. In this framework, intelligence is no longer evaluated solely by algorithmic convergence or statistical predictive accuracy, but by the agent's capacity to maintain dynamic stability, reject physical disturbances, and coordinate multi‐jointed mechanical motion while physically engaging with non‐stationary and uncertain environments [51, 108].

The transition to EI introduces the necessity of managing complex kinematics and rigid‐body dynamics that purely virtual models may simplify. To successfully execute a computationally generated policy, an embodied system must solve inverse kinematics and inverse dynamics in real time, calculating the exact joint torques and angular velocities needed to achieve a desired spatial trajectory under varying load conditions. This necessitates the implementation of advanced control frameworks, such as impedance control and admittance control, which regulate the dynamic relationship between the robotic manipulator and the environment [109, 110]. By actively modulating apparent inertia, damping, and stiffness, EI allows systems to execute compliant manipulation, transitioning from rigid position‐control to force‐aware interaction. This active compliance is critical for mitigating the “sim‐to‐real” discrepancy, as it enables the physical hardware to absorb unmodeled contact forces and geometric tolerances that would otherwise degrade task execution [53].

From an information‐processing perspective, EI is characterized by the continuous and computationally intensive requirement of cross‐modal conversion [111]. Unlike AI, which operates often on pre‐digitized, normalized, and batch‐processed datasets, EI must actively digitize physical reality synchronously with its temporal evolution. During the perception phase, embodied systems capture continuous analog signals—such as mechanical force, thermal gradients, spatial coordinates, and optical variations—through distributed multi‐modal sensor arrays. These high‐dimensional physical interactions are subsequently subjected to analog‐to‐digital (A/D) conversion, introducing system‐dependent quantization errors and signal discretization. To reconstruct a coherent state estimation from these noisy and discrete sensory inputs, EI relies heavily on complex sensor fusion algorithms, such as extended Kalman filters or particle filters. Subsequently, during the execution phase, localized control algorithms compute necessary dynamic compensations and transform these digital commands back into continuous analog signals via digital‐to‐analog (D/A) converters for electro‐mechanical actuation. This bidirectional conversion mechanism enables EI to handle stochastic environmental perturbations, yet it can limit the fidelity of the physical interaction according to the resolution and sampling frequency of the discrete electronic components [51].

Despite successfully enabling physical execution and environmental interaction, EI remains dependent on the architecture and performance of its sensing–computation–actuation loop. In many EI implementations, intelligence is deployed onto the physical hardware rather than being intrinsically built into the material substrate itself [112]. The system commonly integrates discrete sensory modules, data transmission buses, onboard or distributed computational processors, and discrete mechanical actuators. This organization means that many environmental adaptations involve a complete, cyclical process of signal acquisition, network routing, algorithmic inference, and mechanical response [113]. Consequently, the architecture introduces implementation‐dependent transmission latency and computational overhead, which influence the system's operational control bandwidth. According to the Nyquist–Shannon sampling theorem and the fundamental limits of closed‐loop control, the maximum frequency of a physical disturbance that the system can actively reject is partly limited by this total loop delay.

This latency bottleneck becomes critically apparent when the system is subjected to high‐frequency physical disturbances, high‐speed collision dynamics, or complex multi‐physics coupling that significantly outpaces the sampling rates of electronic sensors and the processing bandwidth of local controllers. Furthermore, attempting to scale EI to achieve micro‐level physical adaptation or widespread distributed autonomy exposes practical limitations [114]. Increasing the degrees of freedom in an embodied system can require substantial increases in sensory density, wiring complexity, and computational power. The resulting computational burden of calculating high‐dimensional Jacobian matrices for hyper‐redundant systems can increase energy consumption, while the physical wiring harnesses introduce parasitic stiffness and increased inertial mass, rendering the systems mechanically cumbersome. These trade‐offs motivate complementary designs in which selected local responses are delegated to morphology or material dynamics, thereby providing a transition toward Physical Intelligence [91].

In manufacturing, the perception–decision–action loop provides an operational criterion for EI: sensing of task‐relevant states must influence subsequent planning or control, and the resulting action must provide new physical feedback. Adaptive manipulation, autonomous inspection, and human–robot collaboration satisfy this criterion when they continually adjust to parts, tools, environments, or human activity; a robot executing a fixed trajectory does not necessarily do so.

### 2.3 Physical Intelligence (PI): Deepening Into Intrinsic Material Response

Complementing electronically mediated sensing, computation, and actuation, Physical Intelligence (PI) represents a complementary form of decentralized functionality in the evolutionary continuum [91, 115]. While EI remains influenced by the inherent latency of sequential sensor‐controller‐actuator loops and the von Neumann architectural separation of memory and processing, PI can reduce reliance on external control for selected local functions. It achieves this by embedding task‐relevant response, memory, switching, or regulation within the structural morphology and physicochemical composition of the substrate [67]. This transition fundamentally redefines the boundaries of intelligent systems: the material itself can contribute simultaneously to sensing, information transformation, and actuation. By internalizing part of the stimulus–response mapping at the material or structural level, PI complements electronically mediated EI and enables localized physical functions.

From an information‐processing perspective, the information flow within PI is often continuous, implicit, and deeply coupled with the material state, representing a shift from explicit digital representation toward thermodynamic and mechanical information [91]. In comparison with AI and EI, which rely on the explicit digitization of physical phenomena into discrete bits for algorithmic processing, PI can transform information synchronously with physical evolution. Information is physically encoded within the state of the material or structure—manifesting as stress‐strain distributions, chemical potential gradients, thermal dissipation profiles, or electromagnetic variations. Consequently, selected PI functions may occur without explicit analog‐to‐digital conversion, semantic decoding, or centralized algorithmic inference. The physical deformation, phase transition, or molecular realignment of the material constitutes both the direct physical response to external stimuli and a native form of non‐linear mechanical computation, often formalized as physical reservoir computing [54]. Here, the governing differential equations of the material dynamics contribute directly to the mapping between stimulus, material state, and functional response.

This profound integration of information processing and physical actuation can support rapid, localized responses without routing every event through an external electronic control loop. The relevant timescale nevertheless depends on the mechanism and operating conditions: elastic snap‐through may occur rapidly, whereas heat transfer, diffusion, phase transformation, and self‐healing may require seconds, minutes, or longer. When subjected to highly dynamic environmental perturbations—such as instantaneous impact forces, localized thermal shocks, structural micro‐cracking, or high‐frequency vibrations—physically intelligent materials can generate task‐specific responses governed by their designed structure and material dynamics [116]. Representative implementations include multistable mechanical metamaterials that absorb external mechanical work through rapid geometric snap‐through instabilities and topological reconfiguration, acting as intrinsic mechanical logic gates [117, 118, 119, 120, 121]. This provides a concrete example in which stimulus response and physical reconfiguration produce the task‐level outcome of impact mitigation. Furthermore, self‐healing polymers utilize reversible dynamic covalent bonds or supramolecular interactions to autonomously restore structural integrity driven by localized energy perturbations [122]. Here, material‐level self‐regulation supports the outcome of functional recovery. In these systems, adaptive compliance, shape morphing, and operational stability are realized partly through thermodynamic equilibration and energy minimization principles [123].

Crucially, the emergence of PI contributes to the unified intelligence architecture by establishing a material‐level feedback mechanism for the upstream cognitive layers. The physical responses, localized adaptations, and structural evolution generated by PI continuously alter the observable physical states and dynamic boundaries of the environment. These material‐level transformations generate physically grounded observations that can feed back into the AI training loop [124]. By anchoring the computational layer to measured thermodynamic and mechanical behavior, these observations can constrain data‐driven models. They can support hybrid learning strategies that incorporate conservation laws—such as energy, momentum, and mass—within the loss functions of generative architectures [125]. This can reduce the sim‐to‐real discrepancy, improve robustness under out‐of‐distribution conditions, and help assess whether AI‐generated policies are physically feasible. Ultimately, this recursive feedback mechanism can calibrate the centralized cognitive models, fusing abstraction, embodiment, and material adaptation into a closed‐loop intelligence continuum [126].

Operationally, PI is identified in this Review when a deliberately designed transformation of material or structural state directly performs a task‐relevant function, such as sensing, memory, switching, regulation, computation, morphing, or recovery. Such functionality may arise through stimulus response, physical reconfiguration, and self‐regulation, enabling outcomes including shape morphing, impact mitigation, and functional recovery (Figure 1). This distinguishes PI from EI: EI organizes adaptive behavior through recurrent sensing, computation, and actuation at the agent level, whereas PI delegates a defined part of that behavior to the physical substrate itself. The categories can therefore coexist within a hybrid system. Current PI implementations remain largely task‐specific, and their industrial translation depends on fatigue resistance, environmental stability, fabrication consistency, scale‐up, reprogrammability, and lifecycle verification.

Table 1 distinguishes AI, EI, and PI according to where a task‐relevant transformation is performed and how its physical consequences enter the manufacturing loop. The presence of feedback alone is not treated as a new paradigm, and an integrated manufacturing system may combine mechanisms from all three domains.

**TABLE 1:** Operational criteria, representative manufacturing roles, and current evidence for AI, EI, and PI in smart manufacturing.

Domain	Where the function is realized	Manufacturing role and feedback	Current evidence and near‐term need
AI	Task‐relevant inference is performed in digital models, while the physical process is represented through measured data and explicit constraints.	AI supports inspection, prediction, scheduling, optimization, and design. Its outputs reach the process through machines, controllers, or operators, while new measurements support subsequent model updating.	AI is established in several bounded industrial tasks. Further progress depends on transfer across machines and materials, reliable uncertainty estimation, and verification of physical feasibility.
EI	Function emerges from the coupled operation of sensors, control, actuation, body, and environment; physical interaction must influence subsequent behavior.	EI supports adaptive manipulation, force‐aware assembly, autonomous inspection, and human–robot collaboration. The consequences of an action update state estimation and the next control decision.	The enabling technologies are increasingly deployed in structured and sensor‐rich settings. Robust state estimation, safe contact‐rich adaptation, and generalization beyond predefined operating conditions remain priorities.
PI	A designed transformation of material, structural, or interfacial state directly performs part of the task‐relevant function.	PI supports stimulus‐responsive switching, physical reconfiguration, self‐regulation, shape morphing, impact mitigation, physical computation, and functional recovery. The material response provides the local function and can also be measured by upstream models or controllers.	Current evidence is concentrated in task‐specific components and demonstrators. Reproducibility, fatigue resistance, scale‐up, reprogrammability, and lifecycle verification are central to industrial translation.

The most actionable next steps lie at the interfaces between the three domains. At the AI–EI interface, priorities include transferring models across machines and materials while preserving uncertainty estimates, and converting digital decisions into safe and verifiable behavior under contact and process variability. At the EI–PI interface, control architectures and material responses should be co‐designed so that local physical functions remain observable, controllable, and compatible with system‐level objectives. At the PI–AI interface, reproducible characterization and lifecycle measurements are needed to incorporate changes in material state into model updating and design optimization. Progress on these interfaces can connect computational modeling, embodied execution, and emerging material functions without treating them as a simple replacement sequence.

## 3 Artificial Intelligence in Manufacturing Systems

Artificial intelligence (AI) has become a core computational capability in smart manufacturing, extending from process monitoring and optimization to prediction, generation, and decision support across increasingly connected industrial environments [98, 127, 128, 129]. This expansion has been supported by advances in machine learning, deep neural networks, reinforcement learning, generative models, and foundation models, together with industrial data infrastructures such as the industrial internet of things (IIoT), cyber–physical systems, and digital twins [99, 130, 131, 132].

In the framework adopted in this Review, AI refers primarily to computational systems that transform digital representations of manufacturing states into predictions, generated designs, optimization results, or decision policies. Its defining feature is that the dominant representation and inference processes reside in digital computation; physical execution may be coupled to AI outputs but is not itself required for a system to be classified as AI. This operational definition distinguishes AI from embodied intelligence (EI), where intelligent behavior is realized through a perception–decision–action loop with a physical body, and from physical intelligence (PI), where adaptive functionality is partly encoded in materials, structures, or other physical substrates.

Recent developments have extended manufacturing AI beyond conventional prediction and optimization toward generative intelligence, physics‐informed learning, multimodal reasoning, and autonomous planning [133, 134, 135, 136]. These developments can form interfaces with EI or PI when computational outputs are physically realized through embodied perception–action loops or are used to design and encode adaptive functionality in materials and structures [137, 138]. In the absence of such physical realization, however, physics‐informed or generative models remain AI‐level computational capabilities rather than direct evidence of EI or PI.

Accordingly, this section first clarifies the computational role of AI within the AI–EI–PI framework and then critically examines five major application domains: process parameter optimization, defect detection and quality inspection, predictive maintenance and health monitoring, production scheduling and decision‐making, and generative design and process synthesis. Across these domains, we compare the dominant data and methods, validation environments, evidence of industrial maturity, transferability across machines or process conditions, and major failure modes. We then examine the conditions under which generative intelligence can function as an interface from AI toward EI or PI, before distinguishing limitations that can be addressed within computational AI from those that require tighter coupling with embodied feedback or material‐level mechanisms.

### 3.1 AI as the Cognitive Layer of Smart Manufacturing

Artificial Intelligence (AI) serves as a major computational and cognitive layer of modern manufacturing systems, enabling machines and industrial platforms to perceive, predict, optimize, generate, and make decisions across increasingly complex production environments [98, 128]. Compared with automation thatis based primarily on predefined models, rules, or fixed control logic, AI‐enabled manufacturing systems can learn task‐relevant patterns from industrial data and use them to support adaptive and context‐aware decision‐making [113, 130]. Figure 2 summarizes this role as an information‐processing architecture in which heterogeneous observations of the physical manufacturing world are digitized, represented, transformed through computational cognition, and subsequently converted into digital outputs for downstream physical realization.

**FIGURE 2:**
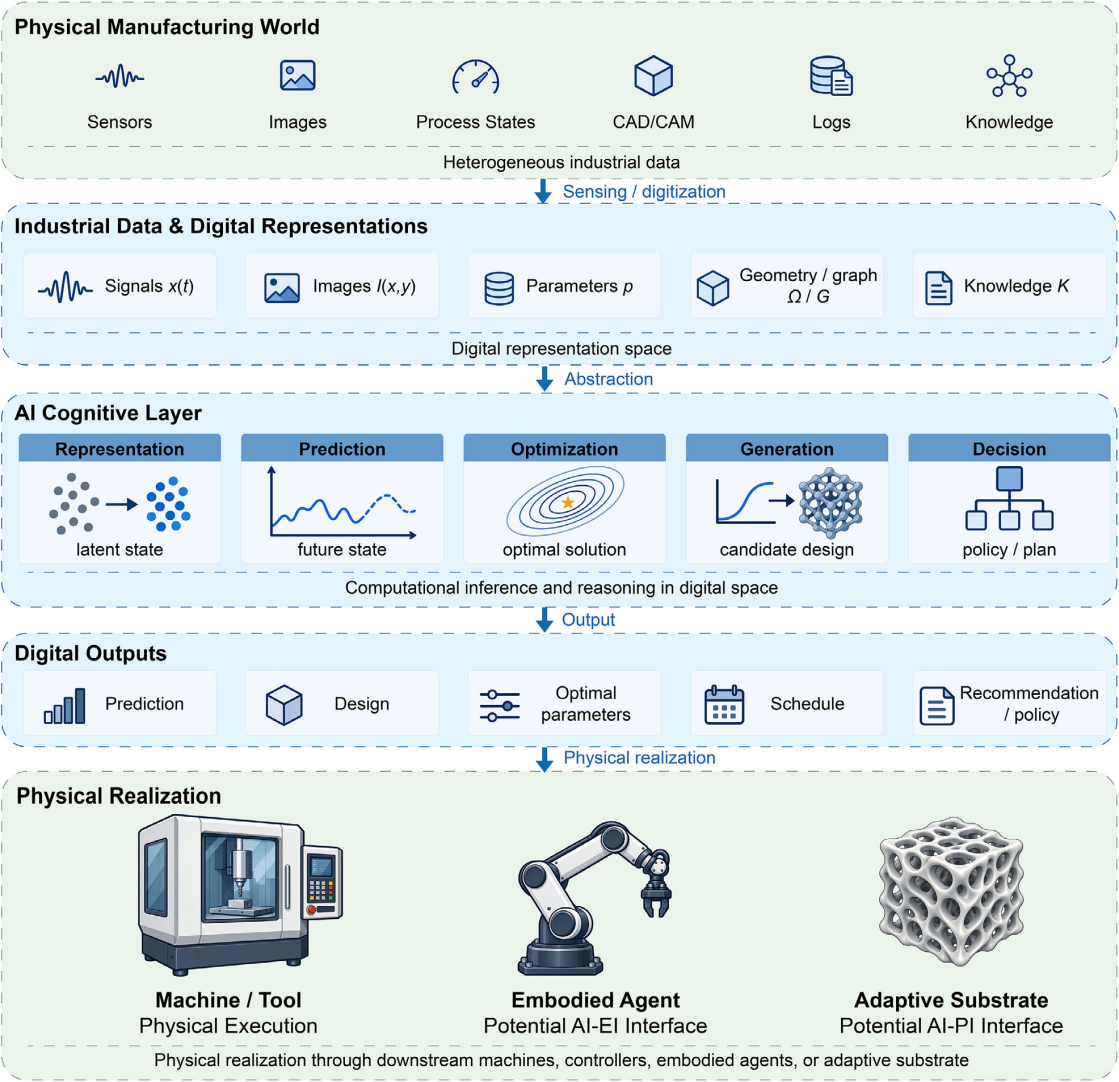
Information‐processing architecture of AI as the cognitive layer of smart manufacturing. Heterogeneous information originating from the physical manufacturing world, including sensor signals, images, process states, CAD/CAM information, operational logs, and encoded knowledge, is first converted through sensing and digitization into explicit digital representations. Within the AI cognitive layer, these representations are transformed through five representative computational functions: representation learning, prediction, optimization, generation, and decision‐making. The resulting digital outputs include predictions, generated designs, optimized parameters, schedules, recommendations, and policies, which can subsequently be realized through downstream machines and tools, embodied agents, controllers, or adaptive physical substrates.

The emergence of this cognitive layer has been driven by the rapid expansion of industrial data ecosystems, spanning multimodal sensing, process, maintenance, and design data [98, 128]. As illustrated in the upper part of Figure 2, physical manufacturing states must first be converted into explicit digital representations, such as time‐series signals, images, process parameters, geometric or graph‐based descriptions, and encoded knowledge, before they can be processed computationally. Through machine learning, deep neural networks, reinforcement learning, and foundation models, AI systems can extract latent correlations and high‐dimensional operational patterns that are often inaccessible to traditional analytical approaches [130, 131, 132, 139, 140]. As a result, AI has progressively expanded its role from isolated process optimization toward a broader range of manufacturing prediction, optimization, generation, and decision tasks, supporting defect diagnosis, predictive maintenance, adaptive scheduling, process synthesis, and decision‐making under uncertainty [134, 141, 142, 143].

From an information‐processing perspective, AI primarily operates within symbolic and data‐centric spaces. Industrial phenomena are first converted into digital representations through sensing and data‐acquisition pipelines, after which computational models perform abstraction, inference, optimization, generation, and decision‐making based on these representations [113, 128, 144]. The AI cognitive layer in Figure 2 makes this distinction explicit by separating the digital representation space from the subsequent computational transformations of representation, prediction, optimization, generation, and decision‐making. The resulting intelligence is therefore fundamentally representational: AI does not directly manipulate physical reality, but instead reasons over encoded approximations of physical processes. Even when AI is embedded within a closed‐loop manufacturing system, its defining inference and optimization processes remain primarily computational rather than intrinsic to the physical substrate. This abstraction capability constitutes a major strength of AI, enabling efficient exploration of high‐dimensional solution spaces and scalable optimization across complex manufacturing systems [95, 145, 146].

Within the broader framework of intelligent manufacturing, AI can therefore be interpreted as the cognitive and abstraction layer of the comparative AI–EI–PI framework. AI focuses predominantly on computational reasoning, virtual optimization, and digital prediction, generation, and decision‐making [137, 138, 147]. As shown in Figure 2, these computational processes typically produce digital outputs such as predicted states, candidate designs, optimized process parameters, schedules, recommendations, or control policies. Such outputs generally require downstream machines, controllers, embodied agents, or adaptive material systems for realization in the physical world [116, 135, 148]. The physical execution of an AI‐generated output should therefore be distinguished from the computational cognition that produced it.

This cognitive role positions AI as a computational intermediary between industrial data acquisition and downstream manufacturing decisions or actions. A representative AI‐enabled manufacturing information‐processing chain therefore proceeds from physical‐state acquisition and digitization to digital representation, abstraction, prediction, optimization, generation, decision‐making, and ultimately physical realization, as summarized in Figure 2. In this pipeline, AI converts raw industrial observations into actionable operational knowledge [98]. Importantly, the bottom layer of Figure 2 also clarifies the boundaries between AI and the other intelligence modes considered in this Review. An AI output coupled to a machine or tool remains primarily an AI‐enabled manufacturing action when the intelligence‐producing transformation is computational. A potential AI–EI interface arises when an embodied agent physically executes computational decisions and the resulting interaction subsequently modifies perception, planning, or behavior. Likewise, a potential AI–PI interface arises when computationally generated designs or policies are physically instantiated such that material or structural states themselves perform part of the task‐relevant adaptive function. From this perspective, AI, EI, and PI represent complementary locations and mechanisms of intelligence that can be integrated across algorithms, embodied agents, and physical matter [116, 117, 138].

### 3.2 Application Fields of AI in Manufacturing Systems

AI applications in manufacturing–including process optimization, defect detection and quality inspection, predictive maintenance, production scheduling, and generative design–have already been extensively summarized in established reviews [106, 149, 150, 151, 152, 153, 154, 155, 156]. Rather than reproducing these method‐ and application‐centered taxonomies [99, 130], we use these representative domains only to clarify the computational role of AI within the broader AI–EI–PI framework and to identify where digital inference begins to interface with embodied interaction or physically instantiated functionality.

Figure 3a summarizes this established landscape through five representative application domains spanning perception, prediction, optimization, generation, and decision‐making. Figure 3b–f provides representative examples. The discussion below is therefore intentionally selective: it focuses on the boundary between computational AI and physical realization, the conditions under which feedback becomes relevant to EI, and the cases in which AI‐generated designs can contribute to PI, rather than repeating comprehensive surveys of individual algorithms or application areas.

**FIGURE 3:**
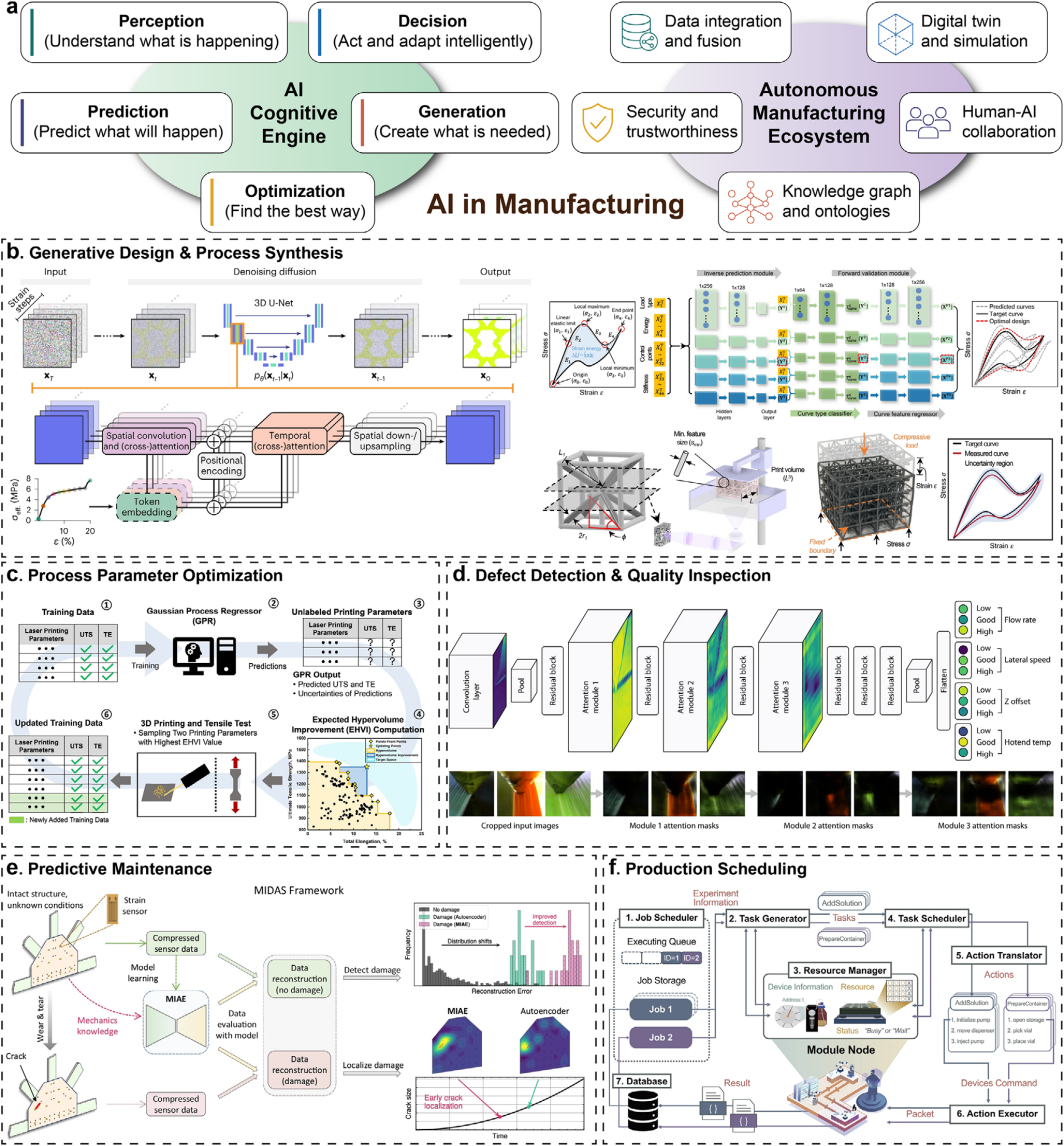
Artificial intelligence in manufacturing systems. (a) Conceptual architecture of AI‐enabled manufacturing intelligence. (b) Generative design and process synthesis, where generative models map target behaviors or design specifications to manufacturable structures and functional material architectures (Reproduced under CC BY 4.0 [136, 157]. Copyright 2023, The Author(s)) (c) Process parameter optimization using surrogate modeling, Gaussian process regression, active learning, and experimental feedback (Reproduced under CC BY 4.0 [158]. Copyright 2025,The Author(s)) (d) Defect detection and quality inspection using machine‐vision models for automated error recognition and process monitoring (Reproduced under CC BY 4.0 [159]. Copyright 2022, The Author(s)) (e) Predictive maintenance and health monitoring using latent health indicators and damage‐localization information (Reproduced under CC BY 4.0 [160]. Copyright 2024, The Author(s)) (f) Production scheduling and decision‐making using AI‐based schedulers for job, resource, and task coordination (Reproduced under CC BY 4.0 [161]. Copyright 2024, The Author(s)).

Generative design and process synthesis illustrate the expanding role of AI from prediction toward the creation of candidate structures, materials, processes, and operational strategies [95, 162]. Current studies increasingly incorporate manufacturability, physical constraints, and process feasibility into topology optimization, inverse material design, process‐aware geometry generation, toolpath planning, and multifunctional structure synthesis [135, 148, 163, 164, 165, 166, 167, 168, 169, 170]. Bastek and Kochmann [136], for example, generated cellular metamaterials conditioned on prescribed nonlinear mechanical responses (Figure 3b), while Ha et al. [157] linked inverse prediction to CAD generation, fabrication, and experimental validation (Figure 3b). These studies reflect a broader shift from geometry generation toward design–process–behavior coupling [136, 171], including structures whose fabricated morphology can support adaptive or material‐level functionality [116, 117]. Nevertheless, generative AI remains computational unless its outputs are physically realized: an AI–EI interface requires embodied execution and feedback, whereas an AI–PI interface requires the fabricated material or structure itself to perform part of the task‐relevant function [138]. This distinction is developed further in Section 3.3.

Process parameter optimization remains a mature AI application, covering machining, additive manufacturing, thermal management, process‐window exploration, and multi‐objective performance improvement [172, 173, 174] [175, 176]. These problems are typically characterized by nonlinear process–property mappings, expensive experiments, and competing objectives, motivating data‐driven surrogates and related learning strategies [99, 171, 177]. Regression, surrogate modeling, Bayesian optimization, and reinforcement learning provide complementary routes for efficient exploration [145, 146]. A representative example is the Pareto active‐learning framework of Lee et al. [158], which combined Gaussian‐process surrogates with targeted experiments for laser powder bed fusion (Figure 3c). Reinforcement learning can further support adaptive closed‐loop optimization [147], but the conceptual boundary remains important: offline or periodically updated parameter optimization is still primarily AI, whereas an EI interface emerges when ongoing physical interaction continuously modifies subsequent decisions or actions. Such systems must also be distinguished by validation level, because process‐specific models may require recalibration under changing machines, materials, or operating windows [125].

Defect detection and quality inspection similarly exemplify AI as a computational interpretation layer that maps heterogeneous observations to manufacturing‐state estimates [134, 141]. Applications include surface defects, cracks, weld quality, dimensional errors, microstructural features, and in‐situ additive‐manufacturing monitoring [178], where variable defect morphologies and sensing conditions complicate deterministic inspection [125, 159]. Recent systems employ CNNs, Transformers, multimodal learning, and vision–language models [179]; CNN‐based methods remain effective for local visual features [130, 141], while Transformer and multimodal architectures improve contextual and cross‐modal reasoning [134, 137, 180, 181]. Brion and Pattinson [159] demonstrated real‐time error recognition and correction across different material‐extrusion conditions (Figure 3d). Multimodal fusion can further connect optical, thermal, acoustic, vibration, and process information [131, 134]. However, richer sensing does not by itself constitute EI: the system remains within the AI layer when sensing terminates in digital classification or prediction, and approaches an embodied loop only when the inferred state alters ongoing physical action and its consequences are sensed again. This distinction is especially important because inspection models remain vulnerable to domain shifts, rare defects, and changes in sensing or process conditions [159, 179, 181].

Predictive maintenance and health monitoring use AI to infer degradation, anomalies, faults, tool wear, and remaining useful life from evolving operational data [182, 183]. Their temporal nature has motivated recurrent, Transformer‐based, graph, and related sequence‐learning approaches [142, 184]; recent models improve long‐range temporal representation [185, 186] and relational reasoning across interconnected equipment [131, 142, 187]. Of greater relevance to the present framework is the growing use of mechanistic priors and physics‐informed learning [125, 133]. Physical constraints can improve data efficiency or consistency, but a physics‐informed model remains AI as long as the task‐relevant inference is digitally implemented; it becomes an AI–PI interface only when the computational output is coupled to, or used to design, adaptive functionality in the physical substrate. Deployment also remains sensitive to sensor drift, changing operating regimes, and scarce failure data [142, 183]. Li et al. [160], for example, combined compressed strain measurements with structural‐mechanics information for automated damage detection and localization (Figure 3e), illustrating how physical knowledge can strengthen computational inference without by itself constituting PI.

Production scheduling and decision‐making represent AI at a higher level of abstraction, where the central problem is the coordination of jobs, resources, machines, and production flows under dynamic uncertainty [143, 188]. Typical formulations include job‐shop scheduling, dynamic resource allocation, and production planning, whose combinatorial complexity motivates learning‐based approaches [139, 189]. Reinforcement learning supports adaptive policy learning [143, 188], graph‐based methods capture dependencies among jobs and resources [139, 189], and multi‐agent systems distribute decision‐making across manufacturing units [147]. These capabilities highlight a strength that remains primarily computational: global coordination and long‐horizon planning under changing demands or disruptions [132]. Yoo et al. [161], for instance, integrated scheduling, task generation, resource management, and device execution in the OCTOPUS architecture (Figure 3f). Such coordination becomes relevant to EI only when physical execution and its consequences recursively update subsequent decisions; by contrast, PI is better suited to selected local functions encoded in material or structural response. The complementary allocation of these functions, rather than replacing one intelligence mode with another, is therefore central to the AI–EI–PI framework.

### 3.3 Generative Intelligence for Manufacturing

Generative artificial intelligence has emerged as one of the rapidly developing branches of modern manufacturing intelligence, supporting the data‐driven generation of candidate structures, materials, processes, and operational strategies [95, 135]. Unlike conventional predictive AI, which focuses primarily on classification, regression, or optimization of existing manufacturing states, generative intelligence aims to create new manufacturable solutions under functional, geometric, and physical constraints [136, 148, 165].

For manufacturing, the relevance of generative AI depends not only on digital generation quality but also on whether generated solutions can satisfy physical and process constraints. In manufacturing systems, intelligence is not realized merely through the generation of virtual geometries or abstract parameter distributions, but through the creation of systems capable of reliable physical behavior in real‐world environments. Consequently, generative intelligence is evolving from a purely computational design paradigm toward a manufacturing‐oriented framework that increasingly integrates physical realizability, process awareness, and adaptive functionality [133, 146, 171]. Under these conditions, generative AI can extend beyond design‐space exploration and serve as an interface to embodied or material‐level functionality when its outputs are physically executed or encoded into fabricated structures [137, 138].

#### 3.3.1 From Generative Design to Generative Manufacturing

Traditional generative AI frameworks in engineering and manufacturing were primarily centered on the synthesis of geometry. Early generative design approaches focused on producing topologically optimized structures, lightweight architectures, or performance‐enhanced geometries by exploring high‐dimensional latent design spaces. Although highly effective for accelerating design exploration, these approaches largely treated manufacturing as a downstream realization step rather than an integral component of the generative process itself [95, 165].

Recent advances indicate a fundamental conceptual shift from generative design toward generative manufacturing. In this emerging paradigm, AI systems are no longer limited to generating static geometries alone, but increasingly generate designs intended to realize prescribed physical behaviors under fabrication, material, and operational constraints [135, 171]. The target of generation therefore expands from shape synthesis to behavior synthesis, where structural response, deformation pathways, environmental interaction, and adaptive functionality become integral components of the generative objective [116, 136].

An important capability is the learning of design‐to‐behavior mappings, through which latent or parametric representations are used to generate candidate designs intended to realize prescribed physical responses after fabrication [95, 136]. Unlike traditional design pipelines that optimize isolated geometric descriptors, generative manufacturing frameworks increasingly capture the causal relationships linking structure, material composition, fabrication pathways, and emergent functionality. When physically fabricated, such generated designs may realize functions including compliant motion, adaptive deformation, multistability, or self‐regulation [117, 148].

This shift fundamentally changes the role of AI within manufacturing systems. Rather than functioning solely as a computational optimization engine, generative intelligence increasingly participates in the co‐construction of physical functionality itself. When the resulting designs are fabricated such that morphology or material properties directly contribute to adaptive behavior, part of the resulting functionality can reside in the physical system rather than exclusively in digital computation [116, 138]. Such cases can form an AI–EI or AI–PI interface, depending on whether the generated functionality is realized primarily through embodied perception–action or through material and structural response.

#### 3.3.2 Physics‐Informed Generative Models

Despite the rapid growth of generative AI, manufacturing environments impose constraints that differ fundamentally from those encountered in purely digital domains. Manufacturing‐oriented generative intelligence must satisfy not only statistical optimality but also physical realizability. Generated outputs must remain manufacturable, mechanically stable, process‐compatible, and robust under real operating conditions. Consequently, purely data‐driven generative approaches are often insufficient for manufacturing applications because they may produce geometrically plausible but physically infeasible solutions [133].

Physics‐informed generative models have therefore emerged as an essential direction for next‐generation manufacturing intelligence [125, 133, 146, 190]. These approaches embed governing laws, material constraints, thermodynamic principles, and process‐dependent behaviors directly into the generative process itself. By integrating mechanics, heat transfer, multiphysics coupling, and constitutive relations into learning architectures, physics‐informed models constrain generative exploration within physically admissible regions of design space [171].

Such integration can occur explicitly through physics‐based loss functions, differentiable simulation constraints, and governing equation regularization, or implicitly through hybrid simulation–learning loops that combine numerical solvers with generative neural architectures [133, 146]. By restricting the feasible solution space, physics‐informed approaches can improve physical consistency and data efficiency when the incorporated governing relations are sufficiently accurate. However, their benefits are application‐dependent: incomplete physical models, uncertain boundary conditions, or process‐specific calibration can limit transfer across materials, machines, and operating regimes.

More importantly, physics‐informed generation represents a broader conceptual transition in AI‐enabled manufacturing. Rather than treating physical laws as external verification tools applied after design generation, these frameworks internalize physical constraints directly into the intelligence generation process itself. As a result, physical reasoning increasingly becomes embedded within the generative architecture, enabling AI systems to produce outputs that are not only computationally optimized, but also physically executable [133, 138]. Importantly, incorporating physical laws into a digital model does not by itself constitute physical intelligence: these methods remain within the AI layer unless adaptive functionality is subsequently realized in the physical substrate.

#### 3.3.3 Generative Co‐Design of Materials, Structures, and Processes

The emergence of generative manufacturing intelligence is also driving a transition from isolated design optimization toward integrated co‐design of materials, structures, and fabrication processes. In conventional manufacturing pipelines, material selection, structural design, and process planning are often treated as sequential and weakly coupled stages. However, many advanced manufacturing systems exhibit highly nonlinear interdependencies among geometry, material composition, fabrication pathways, and functional performance, making such separation increasingly inadequate [135, 171].

Generative co‐design frameworks address this challenge by simultaneously optimizing multiple physical domains within unified AI‐driven architectures. Recent studies have demonstrated that generative models can autonomously discover topologically optimized architectures that exploit structural instabilities, nonlinear deformation, and mechanical anisotropy to achieve adaptive physical responses [136, 165]. In additive manufacturing, generative strategies further enable process‐aware design and adaptive toolpath generation, allowing fabrication parameters and structural functionality to co‐evolve throughout the manufacturing process [135, 146].

At the material–structure interface, generative co‐design frameworks increasingly optimize microstructure, material composition, geometry, and process conditions simultaneously to induce higher‐order functionalities such as self‐actuation, multistability, programmable deformation, and energy‐efficient response [116, 148, 191, 192]. This trend is particularly visible in emerging areas such as mechanical metamaterials, architected matter, and 4D‐printed systems, where physical behavior is deeply coupled to manufacturing history and material architecture.

Importantly, some generative co‐design frameworks target structures whose fabricated material and geometric organization can directly contribute to sensing, deformation, adaptation, or other state‐dependent physical responses [116, 138]. When such functionality is experimentally realized in the fabricated substrate, these approaches provide a potential AI–PI interface in which computational design and physical response are explicitly coupled.

#### 3.3.4 AI as the Translational Layer toward EI and PI

Generative intelligence can form an interface between AI and EI or PI when computational outputs are subsequently realized through embodied action or encoded into adaptive material and structural behavior. In this broader framework, AI serves as the upstream cognitive engine that translates industrial data into executable strategies for embodied systems and physically adaptive materials [137, 138].

Toward embodied intelligence, AI provides the computational foundation for adaptive control, robotic execution, perception‐driven planning, and digital twin cognition. AI models transform heterogeneous industrial data into operational decisions that can be physically enacted through robots, collaborative manufacturing units, and cyber–physical systems [113, 137, 147]. Reinforcement learning, multimodal reasoning, and generative planning architectures increasingly enable manufacturing agents to perform adaptive interaction under uncertain and dynamic production environments. In this sense, AI supplies the abstract cognitive capabilities that drive embodied execution [131, 139].

Toward physical intelligence, AI increasingly contributes to material inverse design, process‐aware physical adaptation, and physics‐constrained generation. Generative models are now capable of co‐optimizing material composition, geometry, and fabrication pathways to produce structures whose intelligent behaviors emerge directly from their intrinsic physical organization [116, 136, 148]. Here, AI does not merely optimize manufacturing operations externally, but actively participates in embedding functionality into physical matter itself.

However, computational co‐design alone is insufficient to establish EI or PI: interface claims require evidence that generated policies are realized through closed‐loop embodied interaction or that generated structures retain the claimed adaptive function after fabrication and physical testing. Generative intelligence is progressively closing the loop between data, design, manufacturing, and behavior, thereby enabling tighter integration among computational reasoning, physical embodiment, and material adaptation.

### 3.4 Limitations of Current AI Manufacturing Paradigms

Despite the rapid expansion of artificial intelligence across manufacturing systems, current paradigms remain constrained by several fundamental limitations that hinder their scalability, robustness, and physical applicability. Although modern AI frameworks have demonstrated remarkable capabilities in perception, optimization, prediction, and generative synthesis, many existing systems still operate primarily as data‐centric computational tools rather than physically grounded manufacturing intelligence [130]. These limitations become particularly pronounced when AI models are deployed in dynamic industrial environments characterized by uncertainty, material variability, process coupling, and incomplete observability [133, 171].

A central challenge lies in the strong dependence of contemporary AI systems on large‐scale, high‐quality labeled datasets [193]. Manufacturing environments often involve heterogeneous sensing modalities, proprietary production conditions, and process‐specific operational constraints, making data acquisition and annotation both expensive and time‐consuming [98, 128, 194]. In many industrial scenarios, failures and defects occur infrequently, resulting in highly imbalanced datasets and limited representation of rare but critical events. Moreover, acquiring labeled data for complex manufacturing processes frequently requires destructive testing, expert intervention, or costly experimental validation. Consequently, many AI systems remain vulnerable to data scarcity and annotation bias, limiting their deployment in low‐data or rapidly changing production settings [141, 182]. These limitations are primarily computational and data‐related rather than inherently embodiment‐related, and can be partially addressed through self‐supervised learning, transfer learning, active learning, synthetic data, and physics‐informed priors.

Another critical limitation concerns the insufficient generalization capability of current manufacturing AI models. Most data‐driven frameworks are optimized under narrowly defined operating conditions and often exhibit significant performance degradation when transferred across machines, materials, geometries, or process conditions. Variations in sensor calibration, environmental fluctuations, and manufacturing tolerances can induce substantial distribution shifts that challenge the robustness of learned representations [99, 159]. As a result, models trained in one production environment frequently fail to generalize reliably to unseen domains. This issue is particularly problematic in manufacturing systems where adaptability and reproducibility are essential for industrial scalability. Although transfer learning, domain adaptation, and foundation‐model paradigms have partially alleviated this problem, robust cross‐process intelligence remains an unresolved challenge [131, 132].

A further obstacle emerges from the persistent gap between virtual optimization and physical manufacturability. Many AI systems achieve impressive performance in simulation environments yet struggle when translated into real‐world manufacturing pipelines. This sim‐to‐real discrepancy arises because computational models often neglect uncertainties associated with fabrication tolerances, material heterogeneity, thermal fluctuations, nonlinear interactions, and process‐induced defects [146, 171]. Consequently, solutions that appear optimal in latent or simulation spaces may become unstable, infeasible, or non‐manufacturable under physical constraints. This challenge is especially critical for generative manufacturing systems, where the generated outputs must satisfy not only statistical or geometric optimality but also fabrication feasibility, structural robustness, and operational reliability [95, 133]. Bridging this gap requires tighter integration between learning algorithms, process physics, and real‐time manufacturing feedback.

A further limitation is insufficient physical grounding in many current manufacturing AI applications. Existing manufacturing AI systems largely externalize intelligence from the physical substrate, operating predominantly in symbolic and data‐centric spaces rather than through direct physical interaction with materials, environments, and embodied systems [138]. While such architectures excel at abstract computation and pattern recognition, they often lack intrinsic awareness of physical causality, environmental coupling, and material adaptation. Consequently, intelligence remains separated from execution and physical realization. This limitation identifies where EI and PI can provide complementary mechanisms: EI can introduce closed‐loop physical interaction and online action feedback, whereas PI can place selected adaptive functions within material or structural responses [116, 137, 195].

These limitations do not imply that AI must be replaced by EI or PI. Data scarcity, uncertainty quantification, model generalization, and cross‐domain transfer remain primarily computational challenges that can be addressed within AI itself. EI becomes particularly relevant when predictions or policies must be continuously updated through physical perception–decision–action loops, whereas PI offers complementary mechanisms when selected adaptive functions can be encoded directly in materials or structures. Neither EI nor PI, however, automatically resolves data bias, global planning, verification, or system‐level safety. The practical challenge is therefore to determine which functions should remain computational, which require embodied feedback, and which can beneficially be delegated to physical mechanisms.

## 4 Embodied Intelligence in Manufacturing Systems

While AI has significantly enhanced data processing and analytical decision‐making in modern manufacturing, many AI‐centered manufacturing approaches still separate high‐level computational inference from continuous physical interaction and execution. To address the dynamic uncertainties of real‐world production, embodied intelligence (EI) provides a complementary framework in which computational intelligence is coupled more directly with physical perception, decision‐making, and action. In this Review, EI refers to manufacturing systems in which a physically embodied agent couples perception, decision or adaptation, and action through an online perception–decision–action loop with its environment. Digital models, AI algorithms, and digital twins may support this loop, but they are not themselves sufficient to constitute embodiment. Accordingly, the presence of sensors, controllers, and actuators alone does not distinguish EI from conventional closed‐loop automation; the distinction considered here lies in the integration of context‐dependent perception, online decision adaptation, physical interaction, and feedback from the consequences of action. The following discussion therefore organizes manufacturing EI according to the functional stages and interactions within this closed loop rather than treating individual sensing, planning, and control technologies as independent evidence of embodiment. In manufacturing, these mechanisms can appear in autonomous robotic or machine agents with perception–decision–action loops, in human–robot systems where decisions and actions are coordinated through physical interaction, and in cyber–physical systems where digital twins support state estimation, planning, and feedback for physically embodied equipment. The overall architecture of manufacturing EI, illustrating the functional relationships among multi‐modal perception, decision, task planning, physical execution, and feedback, is depicted in Figure 4.

**FIGURE 4:**
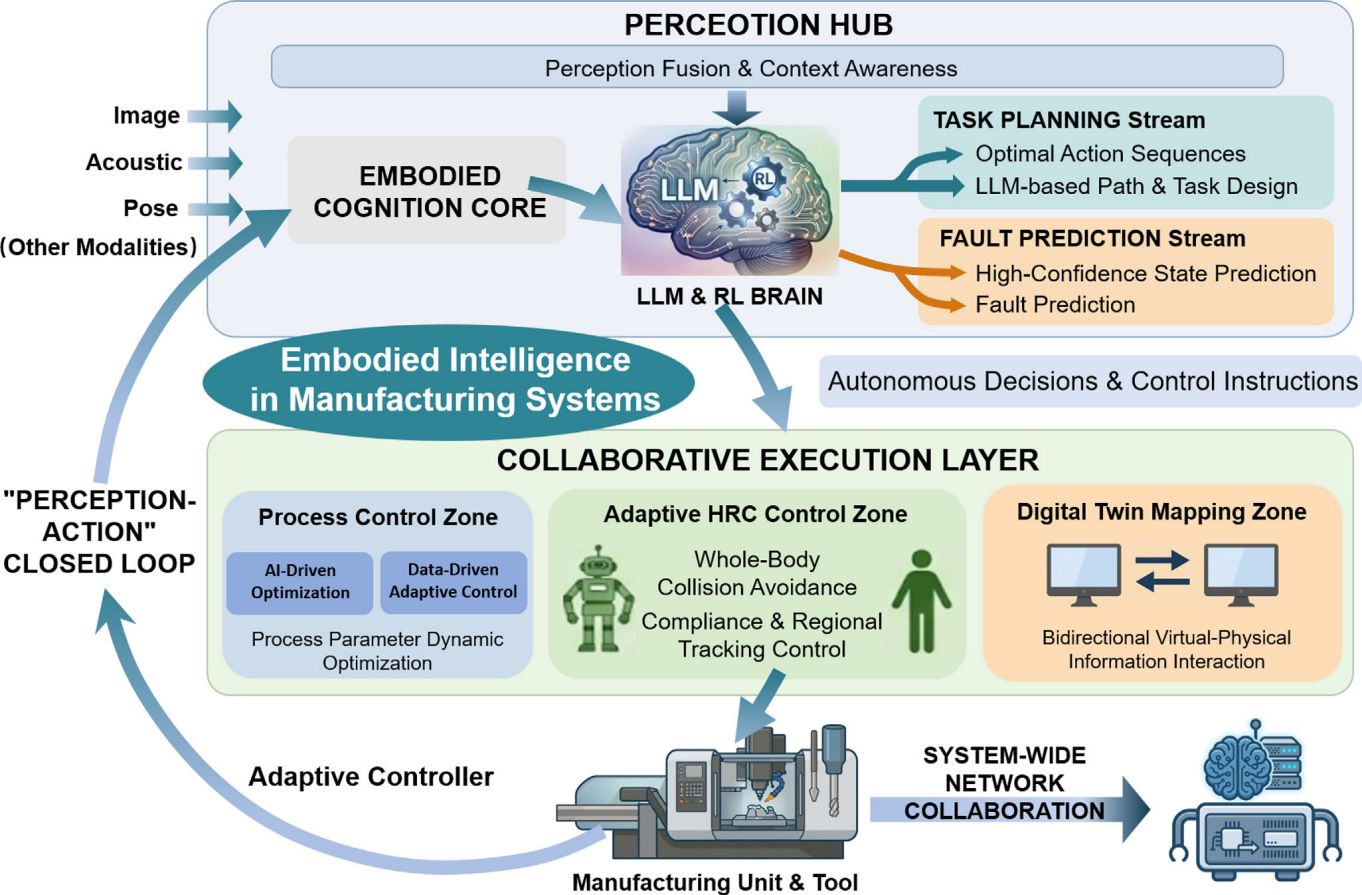
The overall architecture of embodied intelligence in manufacturing systems.

### 4.1 Perception‐Driven Manufacturing Systems

With the development of Industry 5.0, manufacturing research has placed increasing emphasis on human‐centricity, resilience, adaptability, and closer coupling between computational intelligence and physical interaction. The perception–decision–action loop constitutes a useful functional basis for characterizing embodied manufacturing systems. Recent studies by Ren et al. [112] and Xu et al. [196] describe increasing interest in embodied approaches that couple physical interaction with perception, reasoning, and adaptive action.

Embodied agents such as robots or intelligent machine systems integrate sensing, decision‐making, and actuation within a physical platform, while sensors and actuators provide the interfaces through which the agent perceives and acts on its environment. Sun et al. [107] further refined this theoretical framework, emphasizing the integration of perception, cognition, and action through physical interaction. Such interaction allows information acquired during physical execution to update subsequent perception and decision‐making, providing a mechanism for adaptation in changing or partially structured industrial environments.

As a cross‐domain enabling example, the LPAC architecture proposed by Agarwal et al. [197] shows how perception and communication can be jointly modeled through graph neural networks for decentralized multi‐agent coordination in unknown environments. Although this study does not constitute direct manufacturing evidence, the underlying mechanism is relevant to distributed embodied coordination and illustrates how local observations can be incorporated into collective decision processes.

At the perception level, multimodal fusion and in‐situ monitoring technologies can provide richer and more timely state information for adaptive monitoring and downstream decision‐making. To address transient changes in manufacturing processes, single‐dimensional sensing may be insufficient when relevant process states cannot be reliably inferred from a single sensing modality. Li et al. [198] developed a multi‐sensor fusion method based on convolutional neural networks, integrating layer‐wise images, acoustic emission, and photodiode signals to achieve in‐situ quality monitoring of defects in selective laser melting processes (Figure 5e). This study illustrates a typical perception‐level contribution: heterogeneous physical signals are fused into a state representation that can support quality assessment without requiring each sensing channel to be interpreted independently.

**FIGURE 5:**
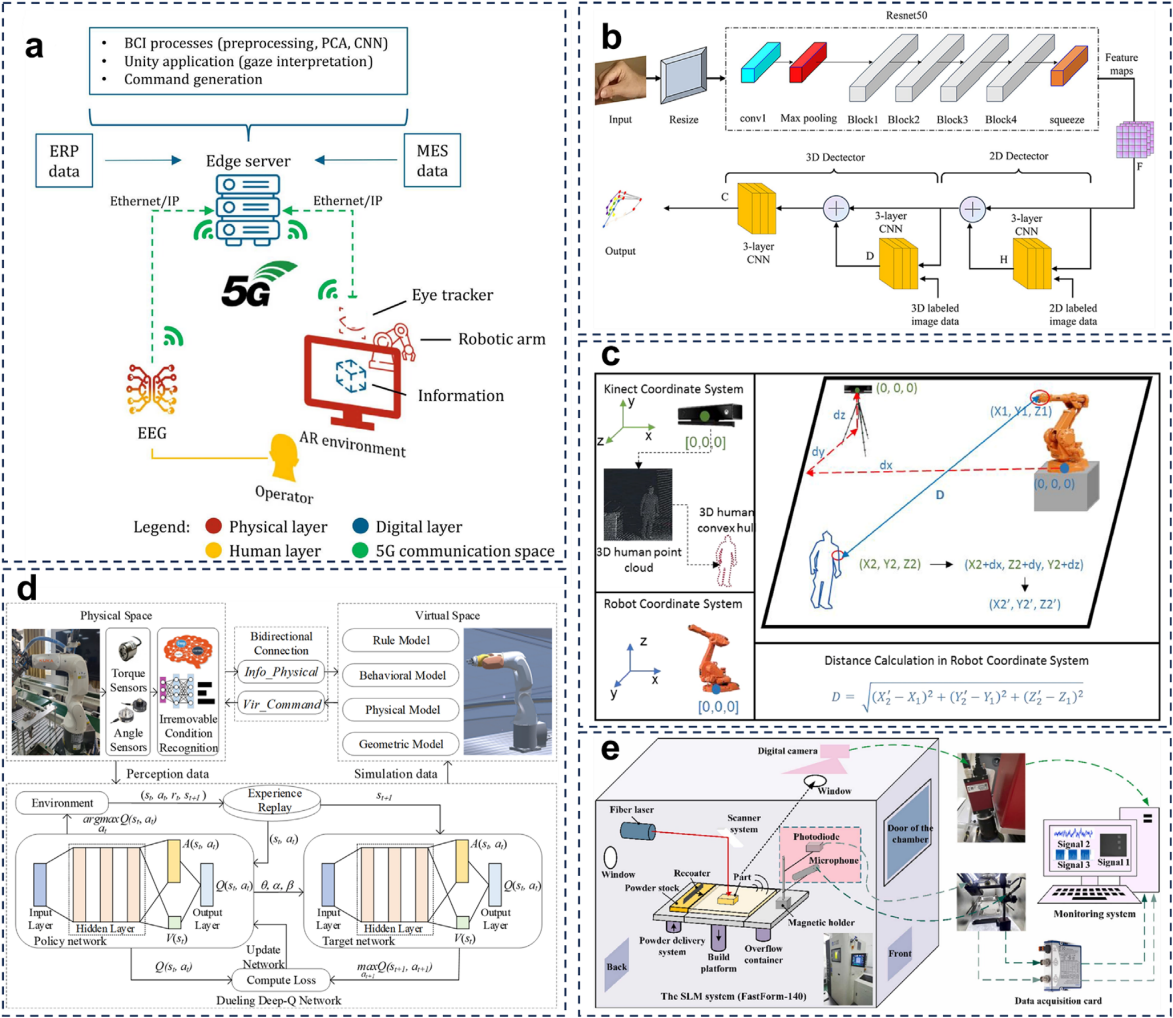
Representative architectures and enabling technologies for embodied intelligence in manufacturing systems. (a) 5G‐enabled mobile communication framework for real‐time human–machine interfaces (Reproduced under CC BY 4.0 [199]. Copyright 2022, The Authors.) (b) Deep learning‐based kinematic perception system for fine‐grained human–robot interaction (Reproduced with permission [200]. Copyright 2024, Elsevier.) (c) Cyber–physical system architecture for proactive safety in fenceless shared workspaces (Reproduced with permission [201]. Copyright 2018, Elsevier.) (d) Digital twin and deep reinforcement learning framework for autonomous dynamic sequence planning (Reproduced with permission [202]. Copyright 2025, Elsevier.) (e) CNN‐based multi‐sensor fusion approach for in‐situ quality monitoring (Reproduced with permission [198]. Copyright 2022, The Society of Manufacturing Engineers).

Building on this, Zhang et al. [111] investigated the integration of multi‐sensor smart tools with digital‐twin‐assisted adaptive control, proposing an adaptive control strategy based on multi‐sensor fusion smart tools. Their study shows that smart tools integrating embedded sensors can not only capture weak signals in real‐time during the cutting process but also support online or near‐real‐time adjustment of machining parameters when combined with AI prediction models. This allows for dynamic optimization of process parameters under tool wear conditions.

This example illustrates how online sensing can update digital representations of manufacturing states and provide timely information for subsequent adaptive decisions. Nevertheless, richer sensing does not automatically produce more reliable embodied perception. Multimodal systems introduce additional requirements for sensor synchronization, cross‐modal calibration, data‐quality monitoring, and robust state estimation when individual sensing channels drift, fail, or become temporarily unavailable. Changes in machine configuration, material condition, tool wear, surface properties, and environmental disturbances may further alter the relationship between measured signals and the underlying physical state. Therefore, the maturity of perception technologies should be evaluated not only by recognition or prediction accuracy under a fixed experimental setting, but also by their ability to maintain reliable state estimates under changing manufacturing conditions.

These studies show that multimodal and in‐situ perception can improve the estimation of manufacturing states under changing process conditions. However, perception alone does not constitute embodied intelligence. Its relevance to EI arises when sensed information is used to update decisions or interaction strategies and when the consequences of subsequent physical actions are fed back into the perception–decision–action loop. The next challenge is therefore not simply to increase sensing dimensionality, but to determine whether the acquired information can reliably alter behavior during ongoing manufacturing operations.

### 4.2 Flexible Robotics and Human–Robot Collaboration

In the context of Industry 5.0, manufacturing research increasingly emphasizes human‐centricity, resilience, and adaptive human–robot interaction. The integration of collaborative systems involving human operators places greater demands on the adaptive capabilities of robots. Rather than treating perception, learning, and control as isolated technical modules, recent advances in human–robot collaboration (HRC) increasingly couple human‐state perception with online planning, interaction adaptation, and physical execution. Within such systems, perceived human, task, and environmental states can influence task allocation, motion planning, interaction strategies, and subsequent physical actions. Such coupling enables robots to modify collaborative behavior in response to changes in human activity, task state, or environmental conditions rather than relying exclusively on fixed task sequences.

Within the perception–decision–action loop, embodied adaptation therefore refers to the online modification of task allocation, motion or interaction strategies, or control policies based on information acquired during ongoing human–robot–environment interaction. This definition is intentionally stricter than simply using a learned model or adaptive controller: the key question is whether evolving interaction information changes subsequent decisions or actions.

At the decision and adaptation level, embodied intelligence can use perceived human and task states to infer intentions, update plans, and adapt collaborative actions during changing or partially structured workflows. Compared with fixed task scripts, recent approaches use online planning, scheduling, learning, and intention prediction to update collaborative decisions during execution. For instance, Darvish et al. [203] proposed an interleaved online task planning framework that integrates real‐time simulations to dynamically reallocate tasks, while Maderna et al. [204] developed flexible scheduling algorithms combined with haptic communication interfaces to adapt resource allocation during sudden failures. To tackle uncertainty in human behavior, reinforcement learning (RL) has been investigated for adaptive task and interaction decision‐making: Zhang et al. [205] utilized RL to optimize task sequence allocation during assembly, and Akkaladevi et al. [206] employed interactive RL to learn collaborative strategies based on human guidance. More recent studies explore Large Language Models (LLMs) for flexible task interpretation and sequential planning in complex manufacturing scenarios [207], together with predictive models that incorporate contextual and interaction information to anticipate human actions [208]. Fan et al. [209] similarly explored LLM‐supported reasoning and path design for industrial robots, illustrating how high‐level generative models may contribute to task interpretation and planning before physical execution. These methods broaden the decision mechanisms available to collaborative robots, but their use does not by itself establish embodied intelligence. In particular, a policy trained or prompted offline remains primarily a computational capability unless information from ongoing physical interaction updates the subsequent plan or behavior. Transferability is also a practical limitation: strategies developed for predefined operators, assembly sequences, workspace layouts, or robot platforms may not retain their original performance when human behavior, task objectives, or production constraints change.

In collaborative manufacturing, online decision adaptation must also account for safety constraints derived from human state, workspace configuration, and interaction context. Safety‐aware perception and planning can therefore modify robot motion or task decisions before physical contact occurs. On the perception front, Liu and Wang [210] developed a deep learning‐based context‐aware anti‐collision system that identifies human poses with low computational cost, supporting context‐aware collision avoidance beyond fixed distance thresholds. For fine manipulation, Yu et al. [200] proposed a kinematics‐driven system combining deep learning‐based hand pose estimation, forming a preemptive safety boundary (Figure 5b). These approaches demonstrate how human‐state perception can constrain or update robot behavior before contact, although the resulting safety performance remains dependent on perception reliability, latency, and the completeness of the modeled workspace.

At the physical interaction level, control algorithms regulate motion, force, and energy exchange between humans, robots, and manipulated objects. Yao et al. [211] proposed a sensorless adaptive admittance control for unknown physical interaction dynamics, while Wang et al. [212] designed a whole‐body collision avoidance controller using quadratic programming to balance soft and hard task priorities. Addressing complex disturbances, Li et al. [213] further developed a robust regional tracking controller based on uncertainty estimators to support trajectory execution during compliant physical contact. Adaptive manufacturing control provides additional enabling mechanisms for this action stage. Liu et al. [214] proposed an adaptive controller based on a gradient‐descent B‐spline wavelet neural network for trajectory tracking under nonlinear actuator dynamics and saturation constraints. Li and Hou [215] developed a Distributed Model‐Free Adaptive Control (DMFAC) scheme in which input/output data are used for disturbance‐rejecting consensus control without requiring an explicit plant model. These methods are relevant to the adaptive execution of embodied systems, but they should be distinguished from complete EI demonstrations because adaptive control at the servo or consensus level does not necessarily include context‐dependent perception or task‐level decision adaptation.

Physical adaptability in HRC can also depend on the compliance and mechanical characteristics of the embodied platform and on how these properties interact with the control strategy. Morphological flexibility is primarily realized at the control‐hardware intersection; for instance, Zeng et al. [216] developed a rigid‐soft controller integrating neural network terminal sliding mode control with adaptive admittance mapping, supporting compliant response under external impacts. Human‐specific adaptation can additionally incorporate physiological information. Slade et al. [217], for example, investigated electromyography (EMG) signals for characterizing three‐dimensional manipulation intentions and adapting interaction‐device characteristics to individual users. Such signals provide another route through which the physical state of the human collaborator can influence the behavior of the robotic system.

HRC therefore provides a representative manufacturing setting in which perception, online decision adaptation, and physical interaction can be coupled within a common loop. Nevertheless, safe contact, compliant control, human‐state prediction, or the use of a learning algorithm alone does not establish embodied intelligence. Many of these techniques originate from conventional robotics and feedback control; their relevance to EI depends on whether interaction‐dependent information continuously influences subsequent decisions and actions. Industrial maturity should therefore distinguish component‐level or task‐specific demonstrations from sustained deployment, where human variability, payload changes, contact uncertainty, communication latency, changing production objectives, and safety certification impose additional constraints.

### 4.3 Embodied Digital Twins

Within embodied manufacturing systems, Digital Twins (DTs) can support the perception–decision–action loop by maintaining synchronized digital representations of physical states, enabling prediction and planning, and returning model‐based information to physical agents. The DT itself is not necessarily an embodied agent; its relevance to EI depends on whether virtual information is bidirectionally coupled with a physical system whose perception, decisions, and actions evolve during operation. Initiating this development, Jiang et al. [218] proposed a multi‐dimensional cognitive framework based on an object‐attribute‐relationship model, supporting multidimensional representation and updating of manufacturing states. Rather than merely reflecting states, this framework integrates human knowledge and machine computing to enrich the information represented in the virtual model. To support state interpretation, Su et al. [219] constructed a three‐layer cognitive DT model based on knowledge graphs, utilizing machine learning to support semantic representation and interpretation of manufacturing states. Rossi et al. [220] further demonstrated a dynamically updated DT framework in which equipment interaction data are collected during operation and used to construct or update the corresponding digital representation alongside its physical counterpart. These studies position state synchronization and model updating as important prerequisites for using DT information within an embodied loop rather than merely for offline visualization.

However, synchronization quality becomes a practical limitation when sensor drift, communication delays, incomplete observations, or model‐update errors accumulate. A digital representation that no longer accurately reflects the physical state may propagate incorrect information into subsequent prediction or planning stages. Consequently, DT maturity in EI should be evaluated not only by geometric or state fidelity at one time point, but also by the stability and timeliness of virtual–physical synchronization during changing manufacturing operations. Beyond state representation, DTs can provide a virtual environment for prediction, evaluation, and planning before decisions are applied to the physical system. Chen et al. [221] highlighted this function in predictive maintenance, using digital models to support earlier prediction of equipment degradation and faults. Within an embodied loop, such predictions can provide additional state information for adapting subsequent maintenance, planning, or control decisions. Enhancing prediction under changing operating conditions, Wang et al. [222] developed a tool wear prediction model that integrates physical mechanisms with transfer learning, incorporating prior knowledge of physical degradation into the learning framework. Such integration can improve the physical consistency of state prediction and provide information for downstream decision‐making, although predictive capability alone does not constitute embodiment. The distinction between prediction and embodied feedback is important. A DT used only for condition monitoring, visualization, or offline forecasting remains an enabling digital model. Its connection to EI becomes stronger when predicted or simulated information modifies a decision that is physically executed and the resulting physical state is subsequently returned to update the model.

The strongest connection between DTs and EI therefore appears when virtual state estimation, prediction, or planning is coupled bidirectionally with physical execution.

Addressing dynamic uncertainties in execution, Liu et al. [202] leveraged the bidirectional coupling between digital and physical systems to achieve information interaction between virtual and real spaces (Figure 5d). Combined with a competitive deep Q‐network, this enabled the DT‐supported system to dynamically generate action sequences based on real‐time perception, supporting adaptive sequence planning at the physical execution level. This type of bidirectional implementation provides stronger evidence for a DT–EI interface than prediction alone because virtual information participates directly in an operational decision loop. Nevertheless, its robustness depends on the fidelity of the digital model, update frequency, network delay, and the degree to which unmodeled disturbances are represented during planning.

Embodied interaction can additionally depend on cyber–physical infrastructure that supports low‐latency communication, distributed state sharing, and remote or safety‐critical operation. Mertes et al. [199] investigated a 5G‐enabled communication framework for mobile human–machine interfaces, while Nikolakis et al. [201] employed a cyber–physical architecture to support proactive safety in fenceless shared workspaces (Figure 5a,c). Liu and Wang [223] further explored digital‐twin‐assisted remote human–robot operation in hazardous manufacturing environments. These technologies provide communication, state‐sharing, and safety infrastructure for embodied interaction, but they should be regarded as EI‐enabling mechanisms rather than embodiment by themselves. At the distributed‐system level, DT‐supported coordination can also extend beyond individual machines or robots. Leng et al. [224] proposed a blockchain smart contract system as a DT for decentralized autonomous manufacturing, combining smart contracts, game‐theoretic interaction, and deep learning to support decentralized coordination under internal disturbances.

Taken together, these studies indicate that DTs can support embodied manufacturing through state synchronization, prediction, planning, communication, and bidirectional virtual–physical feedback. The degree of embodiment should therefore be judged by how DT information participates in the behavior of a physical agent rather than by describing the digital representation itself as an embodied entity.

The maturity of these DT‐enabled embodied loops can vary substantially across functions. State synchronization, monitoring, and predictive modeling can be validated independently, whereas systems in which DT predictions continuously modify physical task policies impose stronger requirements on synchronization accuracy, model validity, communication latency, fail‐safe behavior, and long‐term model maintenance. Accordingly, component‐level DT demonstrations should be distinguished from full‐loop deployment when evaluating industrial readiness.

### 4.4 Closing the Embodied Loop: Feedback, Adaptation, and System‐Level Integration

The functional significance of the perception–decision–action framework emerges when the consequences of physical action are returned to subsequent perception and decision‐making [107, 112, 196]. In such a loop, perception does not merely provide an initial state estimate, and action is not simply the terminal execution of a precomputed command. Physical interaction changes the states of the robot, workpiece, human collaborator, tooling, and surrounding environment, and these changes become new observations that can modify subsequent state estimation, planning, interaction strategies, or control objectives. The embodied loop is therefore characterized by repeated coupling between information acquisition and the physical consequences of action. Figure 6a summarizes this operational criterion: a physical state is perceived and represented as a state estimate, the estimate informs decision‐making or policy adaptation, the resulting action changes the physical system or environment, and the newly generated physical state is observed again to influence subsequent behavior.

**FIGURE 6:**
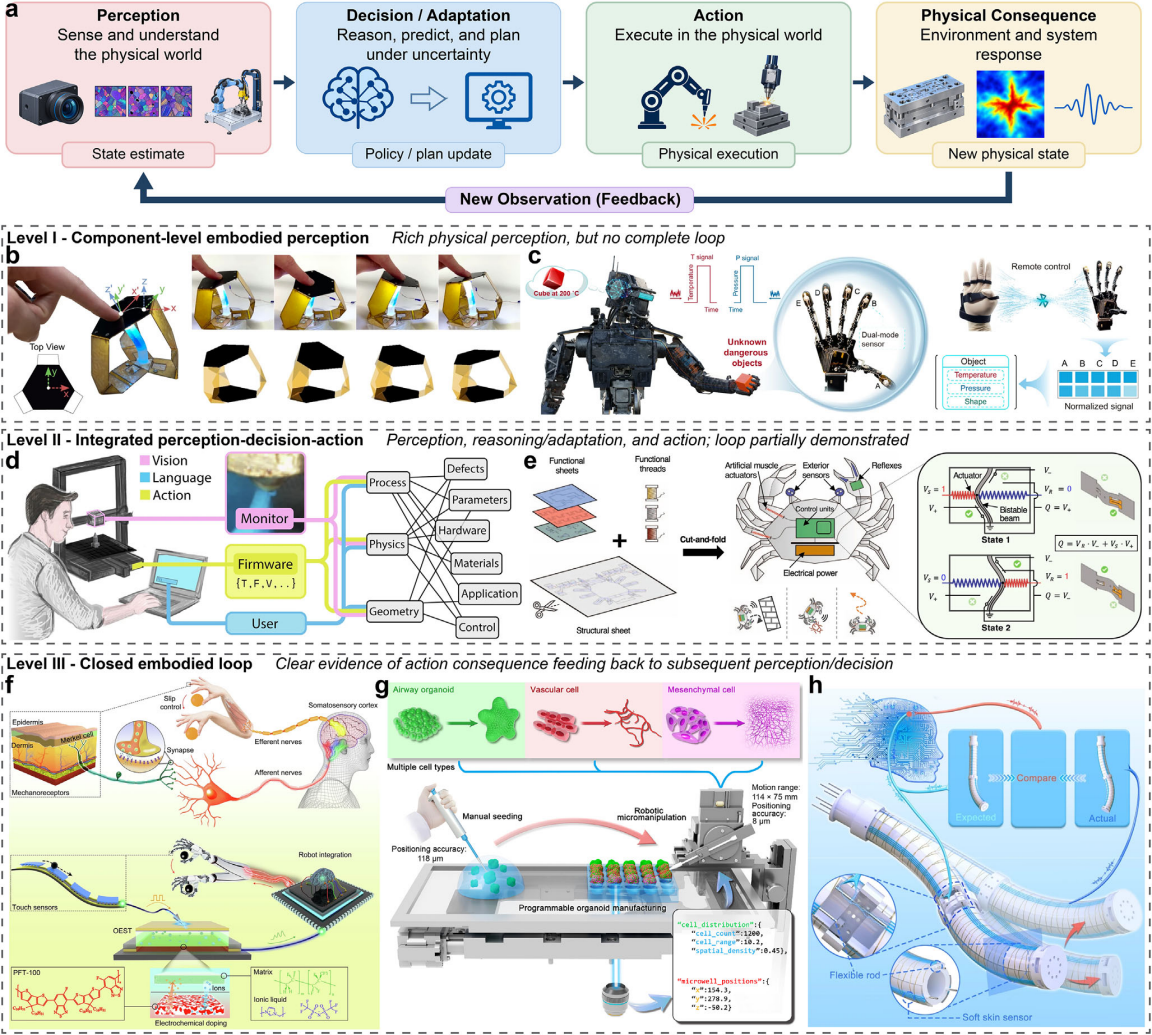
Closing the embodied loop: representative evidence from component‐level physical perception to closed adaptive behavior. (a) Operational criterion for an embodied adaptive loop. Perception generates a state estimate that informs decision‐making or policy adaptation and physical execution; the resulting physical consequence produces a new observable state that feeds back to modify subsequent perception, decisions, or actions. (b, c) Level I: component‐level embodied perception. Representative examples include multimodal deformation and temperature sensing for context‐sensitive machines (Reproduced under CC BY 4.0 [225]. Copyright 2023, The Author(s)), and pressure–temperature tactile sensing for robotic perception under high‐temperature conditions (Reproduced under CC BY 4.0 [226]. Copyright 2025, The Author(s)). (d, e) Level II: integrated perception–decision–action. CIPHER couples manufacturing perception with hybrid reasoning, machine‐instruction generation, and autonomous physical action (Reproduced under CC BY 4.0 [227]. Copyright 2026, The Author(s)), while origami‐based robots integrate sensing, mechanical logic, and actuation to sense, decide, and respond to environmental inputs (Reproduced under CC BY 4.0 [228]. Copyright 2023, The Author(s)). (f–h) Level III: closed embodied loops. Closed‐loop tactile feedback enables slip detection and corrective regrasping (Reproduced under CC BY 4.0 [229]. Copyright 2024, The Author(s)); visual‐servo feedback supports adaptive robotic micromanipulation in organoid biofabrication (Reproduced with permission [230]. Copyright 2025, AAAS); and an expected–actual perception–action loop enables contact detection, target updating, and adaptive motion in a soft continuum robot (Reproduced under CC BY 4.0 [231]. Copyright 2024, The Author(s)).

This interpretation does not imply that feedback control itself is unique to embodied intelligence. Manufacturing systems have long employed sensor feedback to regulate motion, force, temperature, position, and other process variables. The additional emphasis in EI lies in the scope and adaptability of the loop. In conventional feedback control, measured deviations are commonly used to regulate predefined variables around specified references or trajectories. In embodied systems, multimodal and contextual observations may additionally influence task allocation, interaction strategies, planning objectives, or learned policies as the task and environment evolve. The distinction is therefore not simply whether a controller receives feedback, but whether perception, decision‐making, and physical action remain contextually and adaptively coupled across the task hierarchy.

The literature consequently exhibits different levels of evidence for embodied‐loop integration, as summarized in Figure 6. At the first level, individual perception components can provide rich information about physical states without themselves demonstrating a complete perception–decision–action loop. For example, Baines et al. [225] developed a multimodal sensing platform capable of distinguishing bending, compression, stretching, temperature changes, and combinations of these stimuli, thereby providing information‐dense physical‐state perception for context‐sensitive machines (Figure 6b). Similarly, Liu et al. [226] developed a triboelectric tactile sensor capable of jointly perceiving pressure and temperature under high‐temperature conditions and integrated the sensor with a robotic hand and machine‐learning‐based object recognition (Figure 6c). These studies substantially extend the physical states that machines can perceive, but rich perception alone does not establish closure of the embodied loop unless the sensed information is subsequently used to modify physical behavior and the consequences of that behavior are re‐observed.

A second level of evidence emerges when perception is explicitly connected to reasoning or adaptation and subsequently to physical action. Margadji and Pattinson [227], for example, developed CIPHER, a hybrid vision–language–action framework that combines quantitative process perception with physics‐ and knowledge‐grounded reasoning to generate executable machine instructions across multiple manufacturing processes (Figure 6d). This provides a representative manufacturing example in which perceived process states are translated into reasoning and autonomous physical action. Yan et al. [228] further integrated sensing, mechanical information processing, and actuation within compliant origami robots, demonstrating autonomous systems that sense environmental stimuli, implement embedded logic, and generate corresponding physical responses (Figure 6e). Such studies provide stronger evidence for integrated perception–decision–action than isolated sensing or control components. Nevertheless, an integrated sense–decide–act sequence should be distinguished from recursively closed embodied adaptation when the physical consequence of an action is not explicitly shown to update subsequent task‐level perception, decisions, or policies.

The strongest evidence for closing the embodied loop is provided by systems in which the consequences of physical action are explicitly sensed again and alter subsequent behavior. Chen et al. [229] integrated artificial organic afferent nerves with an intelligent robotic gripper and a closed‐loop feedback program: object slippage generates tactile signals, the slip state is recognized, corrective grasping action is initiated, and the resulting stable grasp produces a new tactile state (Figure 6f). This sequence provides a direct example of physical consequence being incorporated into subsequent sensorimotor behavior. In a manufacturing‐related biofabrication context, Tong et al. [230] developed a robotic micromanipulation platform that combines multidimensional cell sensing, visual‐servo control, and adaptive fluid‐dynamics compensation; real‐time observations of seeding‐induced perturbations are used to modify micromanipulation behavior and constrain subsequent cell distributions during programmable organoid fabrication (Figure 6g). Wang et al. [231] demonstrated an expected–actual perception–action loop in a sensorized soft continuum robot, where discrepancies between expected and sensed body configurations enable contact detection and localization, which in turn update subsequent motion during autonomous interaction and navigation (Figure 6h). Collectively, these examples illustrate the defining feature of a closed embodied loop: physical action changes the environment or the body, the resulting state is perceived again, and this new information modifies subsequent action or decision‐making rather than terminating at the first physical response.

Figure 6 therefore spans a continuum of evidence from component‐level physical perception to integrated perception–decision–action and recursively closed embodied behavior. Multimodal sensing and in‐situ monitoring provide state information; planning, reinforcement learning, and human‐intention models support decision adaptation; compliant and safety‐aware control realizes physical interaction; and DTs can provide synchronized state representations, predictive evaluation, and virtual–physical feedback. However, the presence of all these technologies within the same manufacturing system does not automatically demonstrate a complete embodied loop. Evidence of closure requires showing that information generated by physical interaction modifies subsequent system behavior rather than flowing only through a one‐way sensing‐to‐command pipeline.

This distinction is also important for assessing technological maturity. Component‐level sensing or control demonstrations impose substantially weaker requirements than integrated embodied prototypes, while recursively closed manufacturing systems must maintain reliable operation across multiple coupled stages. Perception errors can propagate into planning; planning errors can generate unsafe actions; physical disturbances can invalidate digital models; and delays in communication or model updating can destabilize otherwise effective decision policies. Human–robot applications introduce additional variability associated with operator behavior and safety certification, while DT‐supported systems require continued consistency between virtual and physical states. Accordingly, evidence should distinguish component capabilities, integrated perception–action prototypes, closed adaptive embodied loops, manufacturing‐relevant integrated prototypes, and sustained production deployment rather than treating all systems containing feedback as equally mature. Importantly, the examples in Figure 6 primarily illustrate the first four levels of this progression and should not be interpreted as evidence that sustained industrial deployment has already been achieved across EI technologies.

From the broader AI–EI–PI perspective, EI should therefore be interpreted as a complementary mechanism for physically grounding and updating computational decisions rather than as a replacement for AI or an inevitable intermediate stage toward PI. AI may remain responsible for representation, prediction, generation, and high‐level decision‐making, while EI becomes particularly relevant when these capabilities must be continuously coupled to physical perception and action. PI becomes relevant under a different condition, when selected adaptive functions are implemented primarily through material or structural behavior. The engineering challenge is therefore to determine which functions should remain computational, which require embodied interaction, and which may be delegated to physical mechanisms. In this sense, EI is best evaluated as a system‐level integration problem rather than as a new label for individual sensing, learning, digital‐twin, or control technologies.

## 5 Physical Intelligence in Full‐Life‐Cycle Manufacturing Process

Another fundamental shift in intelligent manufacturing is the transformation from cyber‐centric intelligence to physical intelligence (PI), in which material behavior, process dynamics, and sensing are no longer treated as separate functional layers, but as an integrated biome within the physical entities on the shopfloor [126]. In this scenario, PI in manufacturing is not a mere collection of isolated intelligent technologies, but a full‐life‐cycle intelligence generation mechanism through which physical systems acquire, instantiate, update, and reorganize intelligence across design, fabrication, operation, and evolution at a physical level [232]. Figure 7 summarizes representative PI systems by the embedding of the intelligence, the functions of materials or structures, and current implementations of PI. In this paradigm, PI is identified not by a particular material class or by mechanical computation alone, but by whether a deliberately designed material, structural, or interfacial transformation directly performs a task‐relevant function, such as sensing, regulation, switching, adaptation, memory, recovery, or computation.

**FIGURE 7:**
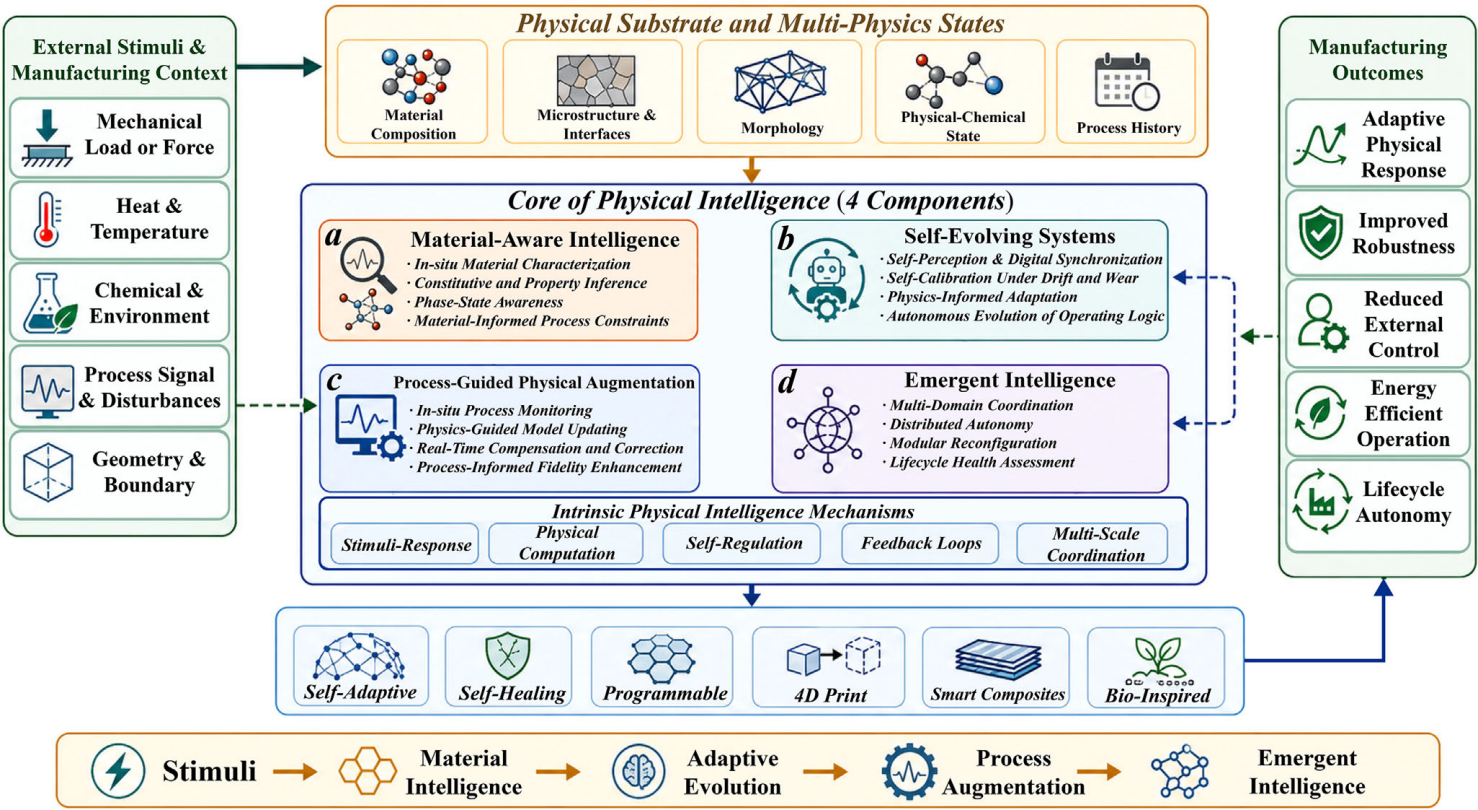
Domain‐level map of physical intelligence (PI) in manufacturing with four fundamental backbones: material‐aware intelligence, self‐evolving systems, process‐guided physical augmentation, and emergent intelligence.

Importantly, stimulus responsiveness alone is not sufficient to classify a material or structure as PI in this Review. We use the term PI when a deliberately designed transformation of material, structural, or interfacial state directly performs a task‐relevant function, such as local regulation, switching, adaptation, memory, recovery, or computation. Smart materials, 4D structures, self‐healing systems, and related responsive platforms should therefore be understood as enabling substrates for PI rather than as PI by definition.

A simple disturbance‐compensation example helps anchor this operational distinction. Consider a manufacturing component subjected to an unexpected mechanical disturbance such as impact, contact, or geometric misalignment. In a conventional sensor‐based architecture, the disturbance is first detected by one or more sensors, converted into digital signals, interpreted by a controller or learning algorithm, and then compensated through commanded actuation. In a PI‐enabled design, by contrast, part of this stimulus–response mapping can be delegated directly to the physical substrate: designed compliance, multistability, snap‐through, stiffness redistribution, or another state‐dependent material or structural response can locally accommodate or attenuate the disturbance before, or alongside, higher‐level electronic control. The distinction is therefore not the absence of AI, sensors, or controllers, but where the task‐relevant transformation is performed. PI can offload selected local and physically constrained responses to matter, while AI and EI retain higher‐level perception, planning, supervision, and reconfiguration.

In the design stage, material behavior, morphology, manufacturability, and process windows set the constraints under which a physically embedded intelligence for certain functions can later be fabricated. But instead of directly constituting intelligence by itself, those constraints set the physical boundaries of a later PI function and the physical boundaries within which intelligent behavior can emerge during the fabrication phase. During fabrication, the designed physical priors are exposed to real process conditions, where thermal history, force interaction, deformation, and sensor observations continuously reveal the mismatch between idealized models and actual material‐process responses. PI is therefore instantiated through this confrontation between planned physical principles and real manufacturing dynamics. In the operation stage, physically instantiated functionality becomes actionable when materials, structures, tools, or components respond to evolving physical states through their intrinsic or programmed dynamics. These responses do not eliminate sensor‐based or algorithmic intelligence; rather, they redistribute selected local, rapid, or energy‐efficient functions into the physical substrate while higher‐level sensing, inference, planning, supervision, and reconfiguration can remain electronically mediated. PI should therefore be interpreted as a complementary mechanism for embedding selected task‐relevant transformations in matter, rather than as a replacement for AI or EI.

Recent experimental demonstrations further show that PI extends well beyond mechanical computing alone. Task‐relevant functionality can be embedded through material‐level sensing and adaptation, morphology‐dependent reconfiguration, structurally integrated control, passive mechanical accommodation, autonomous recovery, or collective interactions among many physical units. These mechanisms span different levels of physical organization, but share a common principle: part of the mapping from environmental or internal state to functional response is carried by the material or structure itself. Representative recent examples are summarized in Figure 8.

**FIGURE 8:**
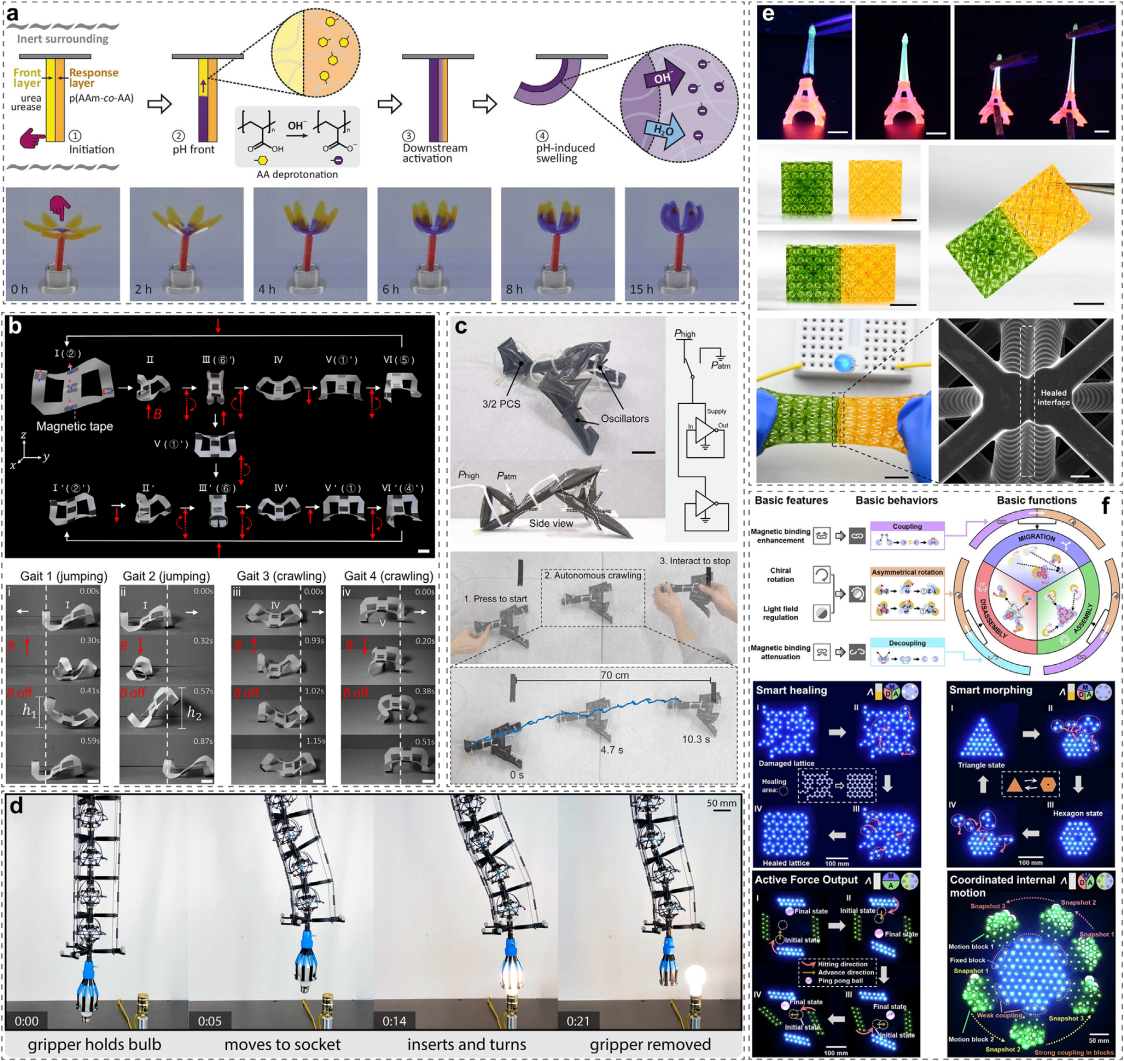
Physical intelligence beyond mechanical computing. (a) Mechano‐adaptive meta‐gels integrate mechanically gated sensing, reaction–diffusion‐based information transmission, and downstream strengthening or actuation within a single autonomous material system (Reproduced under CC BY 4.0 [233]. Copyright 2024, The Author(s)). (b) Multistable thin‐shell metastructures encode multiple mechanically stable configurations that enable reconfigurable manipulation and locomotion through morphology changes (Reproduced with permission [234]. Copyright 2025, AAAS). (c) Soft bistable fabric mechanisms integrate actuation, pneumatic switching, pressure regulation, logic, and oscillation to realize electronics‐free autonomous robotic behaviors (Reproduced with permission [235]. Copyright 2025, AAAS). (d) Mechanical‐metamaterial joints provide selective passive compliance that accommodates geometric misalignment while retaining the ability to transmit torque (Reproduced with permission [236]. Copyright 2025, AAAS). (e) 3D‐printable self‐healing and waterproof ionogels exploit dynamic ionic interactions to autonomously restore damaged or assembled interfaces (Reproduced under CC BY 4.0 [237]. Copyright 2027, The Author(s)). (f) Robo‐Matter uses distributed interactions among reconfigurable robotic building blocks to realize collective adaptation, smart healing, morphing, active force output, and infiltration (Reproduced under CC BY 4.0 [238]. Copyright 2024, The Author(s)).

At the material level, Dúzs et al. [233] demonstrated mechano‐adaptive meta‐gels in which a local mechanical event is converted into a chemical signal, transmitted through reaction–diffusion dynamics, and translated into downstream strengthening or actuation (Figure 8a). At the structural level, Zhou et al. [234] used multistable thin‐shell metastructures to encode multiple stable configurations and thereby enable adaptive manipulation and multigait locomotion through physical reconfiguration (Figure 8b). Yang et al. [235] further integrated bistable actuation and pneumatic switching within a fabric mechanism, allowing physical structure to perform both actuation and control functions in electronics‐free autonomous soft robots (Figure 8c).

The same principle can also complement, rather than replace, computational intelligence. Carton et al. [236] exploited selectively compliant mechanical‐metamaterial joints to passively accommodate manipulation misalignment while a learned inverse‐kinematics model retained higher‐level motion planning, providing a clear example of local physical adaptation operating alongside computation (Figure 8d). He et al. [237] developed 3D‐printable ionogels whose dynamic ionic interactions autonomously restore damaged interfaces and recover sensing functionality in underwater environments, illustrating material‐level recovery embedded directly in the substrate (Figure 8e). At a larger collective scale, Wang et al. [238] demonstrated Robo‐Matter in which interacting robotic building blocks generate emergent functions including adaptation, smart healing, morphing, active force output, and infiltration (Figure 8f). Taken together, these studies indicate that PI spans a continuum from local stimulus–response transformations to structure‐level adaptation and system‐level emergence.

Within this broader spectrum, physical computation represents one important but not exclusive realization of PI. Mechanical neural networks and computing architectures are particularly useful for demonstrating how mapping, memory, logic, classification, or learning can be physically instantiated, but they should be viewed as one subset of a broader PI landscape that also includes local regulation, passive adaptation, reconfiguration, autonomous recovery, and collective physical behavior.

### 5.1 Material‐Aware Intelligence

Material‐aware intelligence is realized in an architected solid whose stiffness, geometry, or stable configuration carries information and executes a mapping [138, 239]. Current progress in this domain focuses on the development of material‐informed frameworks that synchronize in‐situ material characterization with real‐time process control, effectively addressing the challenges posed by material variability and non‐linear constitutive behaviors [240].

At the material and structural levels, recent PI studies have increasingly explored how geometry, constitutive behavior, multistability, and physical state can directly encode task‐relevant mappings. Yasuda et al. [241] mapped mechanical computing onto bistable mechanisms, origami, and wave‐based substrates, so that deformation itself performs logic and memory. Mei et al. [242] built mechanical neural networks with neurons that realize weighted summation and inversion through spring–linkage assembled systems (Figure 9a). Wu et al. [243] classified handwritten digits by routing mechanical signals through a weights matrix of mechano‐synapses in a bistable kirigami metamaterial (Figure 9d). Hopkins et al. [244] used binary‐stiffness beams inside mechanical neural‐network metamaterials, so that discrete stiffness states become learnable weights. Yang et al. [245] computed with transmissive snapping of kirigami shells, so that a snap‐through event is the logic transition. Chen et al. [246] built spatially adaptable mechanical neural networks with multiple fully connected layers, so that the lattice layout is the trainable architecture. These advancements collectively underscore that material‐aware intelligence is not merely about sensing, but about utilizing the intrinsic physical laws of the workpiece to guide the manufacturing evolution.

**FIGURE 9:**
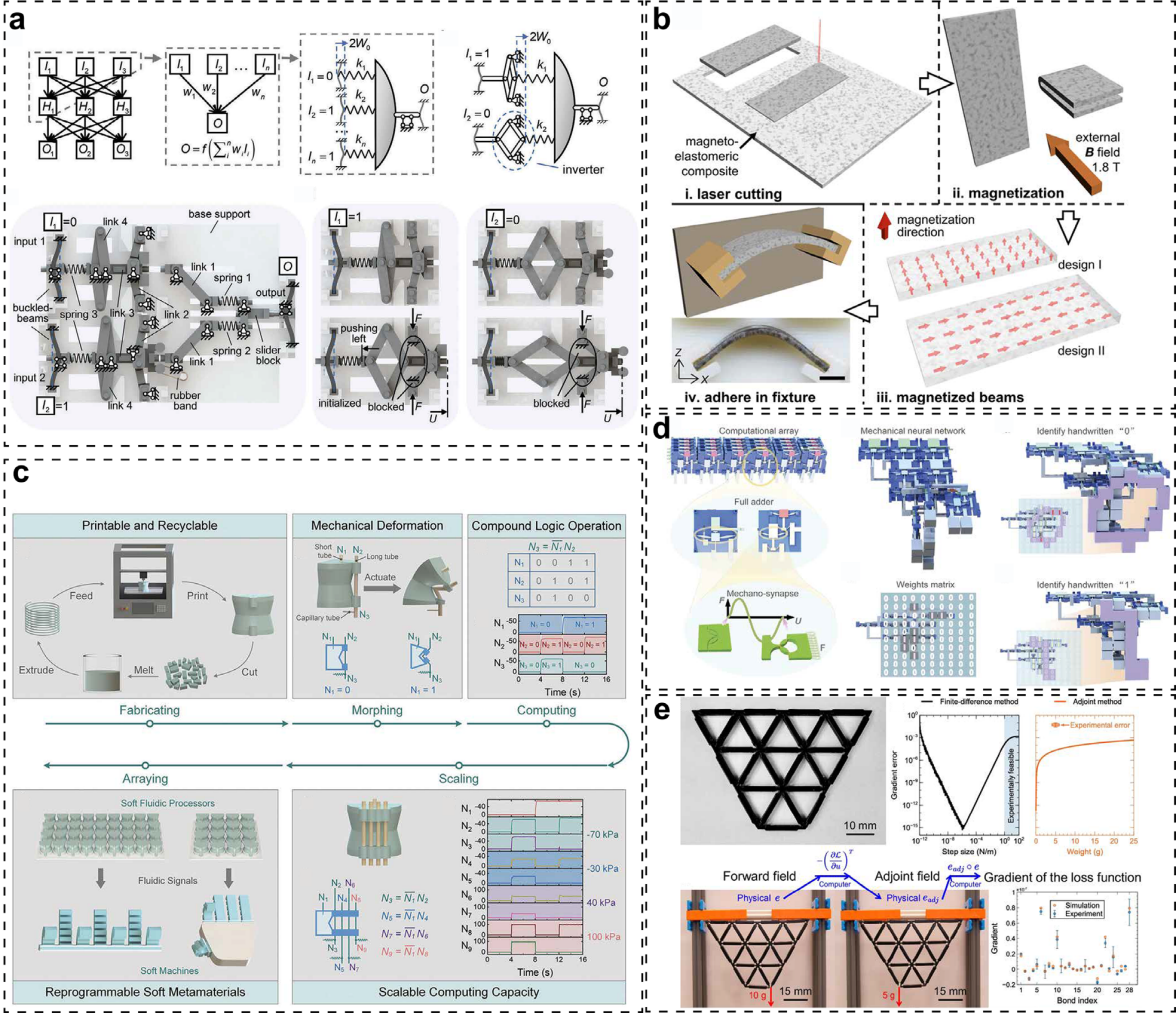
Physical neural networks and mechanical computing hardware in which mapping, memory, programming, classification, and learning are executed by the fabricated solid. (a) Mechanical neural networks with explicit mechanical neurons for weighted summation and inversion (Reproduced under CC BY 4.0 [242]. Copyright 2024, The Author(s)) (b) Magnetically tunable bistable elements for programmable mechanical devices (Reproduced under CC BY 4.0 [247]. Copyright 2023, The Authors.) (c) Reprogrammable metamaterial processors using fluidic logic to control soft machines (Reproduced under CC BY 4.0 [248]. Copyright 2023, The Author(s)) (d) Mechanical metamaterials for handwritten‐digit classification using mechano‐synaptic weights (Reproduced under CC BY 4.0 [243]. Copyright 2023, The Author(s)) (e) A 3D‐printed all‐mechanical neural network trained by in‐situ backpropagation (Reproduced under CC BY 4.0 [249]. Copyright 2024, The Author(s)).

### 5.2 Process‐Guided Physical Augmentation

From the perspective of Physical Intelligence, the manufacturing process functions as a continuous physical truth generator in which the actual evolution of materials, structures, tools, machines, and environments is progressively revealed under real manufacturing conditions. Before fabrication, the intended network can be a designed lattice, bistable beam, kirigami sheet, or fluidic unit with special material properties and process windows. Once the process begins, however, thermal history, force interaction, phase transformation, tool wear, geometric drift, residual stress, and sensor feedback expose the gap between these prior assumptions and the evolving physical reality. In this sense, the manufacturing process becomes a real‐time physics oracle for model correction for it continuously generates the most immediate evidence about how the physical system actually behaves, while computational models remain provisional representations that must be updated according to this unfolding reality.

Process‐Guided Physical Augmentation therefore becomes the manufacturing path that writes physical intelligence into the specific work flow. It represents a paradigm in which manufacturing is an active mechanism for rewriting the model itself. The role of process data is not simply to reduce prediction error, but to convert transient in‐situ phenomena into physically meaningful constraints, state updates, and decision signals. This logic is particularly important in intelligent manufacturing, where sparse sensing, uncertain boundary conditions, nonlinear material behavior, and multi‐physics coupling make it impossible for any offline model to remain fully valid throughout the life cycle. Physics‐informed methods and hybrid mechanistic–data‐driven modeling provide enabling tools for this paradigm by embedding governing laws, residual constraints, and process history into the physical architecture [250, 251, 252]. Through these tools, the process actively augments the physical model, allowing manufacturing systems to maintain interpretability, generalization, and robustness under noisy, incomplete, and evolving industrial conditions.

Existing studies can be reinterpreted through this process‐as‐oracle perspective. Pal et al. [247] laser‐cut magnetoelastomeric sheets and magnetized the beams in a strong field, so the process history sets the programmed magnetic pattern (Figure 9b). Song et al. [253] designed additively manufacturable micro‐mechanical logic gates, so that the printed geometry performs Boolean operations. He et al. [254] assembled electronics‐free soft robots from modular bodies whose distributed control is written into the structured elastomer. Pan et al. [255] fabricated a mechanical‐computing metastructure that converts vibration into a self‐powered sensing and computing response. Yang et al. [256] programmed mechanical logic in bistable soft shells whose as‐built geometry stores the gate function.

Through these studies, Process‐Guided Physical Augmentation thus transforms manufacturing from a passive object of prediction into an active model updater. By treating the factory floor as an in‐situ physical oracle, transient phenomena such as cutting‐force fluctuations, thermal drift, wear progression, deformation, and calibration residuals become the basis for continuously rewriting model fidelity, control logic, and system reliability. This paradigm makes the manufacturing process a central engine of PI formation, linking fabrication‐stage physical instantiation with operation‐stage adaptation and lifecycle evolution.

### 5.3 Self‐Evolving Systems

Self‐evolving systems describe the long‐term adaptive dimension of Physical Intelligence. Thermal drift, structural deformation, material aging, tool wear, vibration, and energy dissipation are therefore treated not only as disturbances to be corrected, but also as informative traces of the system's physical evolution [257]. Over time, these traces can update the system's internal models, process parameters, and configuration policies [258, 259].

The central idea of self‐evolving PI is to turn irreversible physical change into a source of autonomous calibration. Conventional automation usually assumes that the machine, process model, and control strategy remain relatively stable after deployment, with feedback mainly used to correct short‐term deviations. A self‐evolving system instead treats the equipment, workpiece, material state, and process environment as physical entities that continue to change during operation [260]. In this sense, the system is not simply controlled from outside, but actively learns from its own physical transformation, making it possible to sense changing physical states and translate them into adaptation over the life cycle [261].

Existing studies can be read from this perspective. Li et al. [249] trained 3D‐printed all‐mechanical neural networks by in situ backpropagation, with gradients obtained from member elongation (Figure 9e). Moreover, Stern et al. [262] formulated supervised learning in physical networks, so that local physical rules change stiffness or conductivity toward a target map. Dillavou et al. [263] demonstrated emergent learning in a nonlinear analog network that performs inference in the physical hardware. Altman et al. [264] demonstrated coupled learning in elastic networks, so that local strain updates train the mechanical graph. Li et al. [265] trained topological mechanical neural networks as classifiers by in situ backpropagation, with gradients available from the printed members.

Taken together, self‐evolving systems mark a shift from real‐time interaction to life‐cycle physical adaptation. The manufacturing system is no longer only a passive mirror of the physical process, nor only an embodied agent responding to immediate sensory inputs. It becomes a physics‐driven, self‐reconfiguring entity whose intelligence is gradually shaped by its own thermodynamic, mechanical, structural, and material evolution. This long‐horizon adaptation provides the operational link between process‐guided physical augmentation and emergent intelligence. Local physical changes are first sensed and modeled, then accumulated into adaptive behavior, and eventually coordinated across machines, tools, workpieces, and production cells to support system‐level PI.

### 5.4 Emergent Intelligence in Industry

A trending direction within PI is the coordination of multiple physical entities to form system‐level behavior, a systemic state where the non‐linear synthesis of material‐aware, self‐evolving, and process‐augmented layers results in collective capabilities that exceed the sum of individual components. It arises from the interaction of mismatched time scales, physical constraints, and material memory within the manufacturing life cycle. In manufacturing systems, different physical processes do not evolve synchronously, each physical system possesses a unique evolving time scale. These mismatched time scales are further constrained by different governing physical laws that determine what state transitions are physically feasible.

From this perspective, Emergent Intelligence is not the direct output of a single model. Rather, it appears when history‐dependent physical states reshape how the manufacturing system represents, decides, and acts across machines, tools, workpieces, materials, and process environments. Therefore, Emergent Intelligence is not merely an additive property but a physical memory accumulation of the system's ability to coordinate heterogeneous data and multi‐domain physical laws across the entire manufacturing life cycle. By internalizing these cross‐layer dynamics, the system achieves intelligence‐on‐demand, allowing it to navigate unforeseen perturbations and unstructured tasks with high‐level resilience and trustworthiness.

A foundational requirement for Emergent Intelligence is the effective coordination of massive heterogeneous data with complex physical laws, particularly within the Industry 5.0 paradigm. In response, Byun et al. [266] propagated mechanical solitary waves through cascaded logic elements to actuate autonomous soft‐machine prototypes. Jiao et al. [248] built reprogrammable metamaterial processors that execute fluidic logic in printed soft units and used the processor output to control a soft machine (Figure 9c). Li et al. [267] stored high‐density binary states in reprogrammable mechanical computing metastructures whose stable configurations are the memory, opening possibilities for hard‐ware embedded emergent intelligence. Mousa et al. [268] built metamaterials that execute parallel mechanical computing tasks in the same solid. Hu et al. [269] raised computational density in spatially programmable origami networks and demonstrated autonomous path planning in integrated robots. Chen et al. [270] assembled bistable origami switches into a mechanical programmable gate array addressed and activated by a robot.

Collectively, these methods illustrate that Emergent Intelligence is the functional culmination of the PI framework, transforming the manufacturing cell into a physics‐honest entity that anchors its decision‐making in the deep internalization of governing laws.

The four introduced dimensions (Material‐Aware Intelligence, Process‐Guided Physical Augmentation, Self‐evolving Systems and Emergent Intelligence) correspond to different phases of the manufacturing life cycle rather than independent categories. By leveraging the continuous synchronization between physical and virtual layers, the system achieves a state of “distributed autonomy,” where optimal operational strategies emerge organically in response to thermodynamic and environmental fluctuations. Ultimately, such systemic intelligence bridges the gap between idealized models and real‐world implementation, paving the way for a more resilient, efficient, and human‐centric industrial future.

## 6 A Unified Framework: AI–EI–PI Co‐Evolution in Smart Manufacturing

### 6.1 From Layered Intelligence to Co‐Evolving Manufacturing Systems

Conventional paradigms of intelligent manufacturing have largely been established upon a hierarchical interpretation of intelligence, in which AI functions as the centralized cognitive module responsible for prediction, optimization, and decision‐making, embodied intelligence (EI) serves as the executional interface translating computational outputs into physical actions, and physical intelligence (PI) is treated primarily as the passive material substrate through which functionality is ultimately realized [113, 128]. Such an architecture has historically provided a useful abstraction for industrial automation because it enables clear separation between computation, control, and physical implementation. However, this layered interpretation becomes increasingly insufficient for describing advanced manufacturing systems operating under real‐world uncertainty, dynamic environmental perturbations, evolving process conditions, and continuously changing physical constraints [98, 129, 171].

In practical manufacturing environments, intelligence does not emerge through unidirectional transmission from algorithms to machines and subsequently to materials. Instead, manufacturing systems continuously reshape their own operational landscape through recursive interactions among computational reasoning, embodied execution, and physical realization [113, 137, 138]. Material deformation alters sensing conditions and observability; environmental interaction modifies control policies and behavioral adaptation; fabrication processes reshape future learning distributions; and embodied actions introduce new physical constraints that recursively influence computational optimization itself [116, 146, 159]. Under such conditions, manufacturing intelligence can no longer be adequately interpreted as a static hierarchy composed of isolated functional layers. Rather, it increasingly behaves as a dynamically coupled adaptive system in which representation, embodiment, and material response continuously co‐evolve across the manufacturing lifecycle [135, 148].

This transition motivates a broader conceptual reframing of intelligent manufacturing. AI, EI, and PI should not be regarded as independent modules stacked within a top‐down control pipeline, but as mutually interacting subsystems that simultaneously constrain and expand one another. AI provides abstraction, representation learning, predictive modeling, and generative reasoning within symbolic and data‐centric spaces [95, 130, 132]. EI enables physically grounded interaction through sensing, actuation, adaptive control, and environmental coupling [137, 147]. PI embeds intelligence directly into material organization, structural morphology, and intrinsic physical response [116, 117, 138]. Critically, none of these domains operates in isolation. Instead, intelligence in advanced manufacturing emerges from continuous bidirectional coupling between representation (AI), embodiment (EI), and material instantiation (PI), where each domain recursively modifies the operational space of the others [136, 148].

From this perspective, intelligent manufacturing is undergoing a fundamental paradigm transition:

$$\begin{array}{cc} \text{layered automation} & \to \text{closed-loop intelligence}\\ & \to \text{co-evolving manufacturing ecosystems} \end{array}$$

Traditional automation architectures localize intelligence primarily within centralized computational systems, while machines and materials function predominantly as executional components. Closed‐loop intelligent manufacturing partially relaxes this separation by introducing adaptive feedback between sensing, control, and operational response [113, 159]. Yet emerging manufacturing systems increasingly extend beyond feedback‐driven optimization alone. Intelligence becomes distributed across computational abstraction, embodied interaction, and material behavior, such that manufacturing functionality emerges collectively from their mutual adaptation rather than from any isolated intelligence layer [116, 138].

Within this co‐evolutionary framework, AI no longer acts solely as an external optimization engine detached from physical realization. Instead, learning and reasoning become increasingly conditioned by embodied interaction and material dynamics [133, 137]. Simultaneously, EI extends beyond robotic execution to function as an adaptive mediation layer connecting digital cognition with physical environments [137, 147]. PI further transforms from passive material realization into an active contributor to intelligence generation itself, where morphology, compliance, structural instability, and adaptive matter directly participate in sensing, regulation, computation, and response [117, 138, 195]. Consequently, manufacturing intelligence progressively shifts from algorithm‐centered automation toward physically distributed cognition embedded across algorithms, machines, materials, and environments.

Unlike conventional top‐down manufacturing pipelines, the proposed framework emphasizes continuous bidirectional coupling among representation and abstraction space (AI), embodiment and interaction space (EI), and material and physical realization space (PI). Rather than transferring intelligence sequentially from computation to execution, the framework conceptualizes manufacturing as a recursive adaptive ecosystem in which computational cognition, embodied behavior, and physical matter continuously reshape one another across multiple spatial and temporal scales. In this view, intelligence is no longer localized within algorithms alone, but distributed throughout the interactions linking digital models, embodied systems, and adaptive physical substrates [113, 138].

### 6.2 Mechanisms of AI–EI–PI Co‐Evolution

The convergence of artificial intelligence (AI), embodied intelligence (EI), and physical intelligence (PI) cannot be adequately explained through loosely defined notions of interaction alone. Rather, co‐evolution in intelligent manufacturing emerges through a set of tightly coupled adaptive mechanisms that continuously redistribute information, constraints, and functionality across computational, embodied, and physical domains [138]. In this framework, manufacturing intelligence is not generated by a single centralized controller, but arises from reciprocal adaptation among representation, embodiment, and material realization. Consequently, the manufacturing system itself becomes a continuously evolving dynamical entity whose operational behavior is shaped simultaneously by learning processes, embodied interaction, and physical response [113, 116].

#### 6.2.1 Bidirectional Feedback Loops

At the core of AI–EI–PI co‐evolution lies a multi‐loop adaptive feedback architecture in which information continuously circulates between computational models, embodied agents, and physical environments [113, 159]. Unlike conventional manufacturing pipelines that follow a unidirectional sequence from sensing to computation and then execution, co‐evolving manufacturing systems operate through persistent bidirectional coupling across all intelligence domains. Each subsystem not only produces outputs for downstream processes, but also actively modifies the operational conditions under which the other subsystems learn, adapt, and evolve [146].

Within this architecture, AI primarily contributes policy generation, adaptive planning, and control strategy synthesis. Through predictive reasoning and abstraction, computational models transform heterogeneous manufacturing data into executable operational policies that guide embodied systems under uncertain production conditions [130, 143, 188]. These outputs are subsequently instantiated through EI, where robotic agents, adaptive manufacturing platforms, and cyber–physical systems convert abstract computational strategies into physical actions, fabrication trajectories, and environmental interactions [137, 147].

The interaction between EI and PI is particularly important because physical realization is not a passive consequence of execution alone. Embodied systems actively reshape material organization, structural morphology, thermal evolution, and process‐dependent physical states during manufacturing operations [135, 171]. Simultaneously, PI imposes physical constraints back onto EI through compliance, deformation, contact dynamics, environmental coupling, and morphology‐dependent behaviors [116, 117, 138]. As a result, embodiment is continuously regulated by the evolving physical properties of the system itself rather than solely by predefined control policies.

Equally critical are the reverse information pathways through which EI and PI reshape AI. Embodied systems continuously aggregate sensory information, compress behavioral states, and generate real‐time feedback that updates computational representations [134, 159]. More fundamentally, PI directly modifies the statistical structure of the data observed by AI systems. Material adaptation, fabrication variability, thermal fluctuations, wear accumulation, and environment‐dependent physical responses continuously alter the distribution of manufacturing observations, thereby reshaping the learnable representation space itself [125, 171]. In this sense, PI does not function merely as the terminal realization layer of manufacturing intelligence. Instead, it acts as an active generator of physically grounded learning signals that recursively influence future computational reasoning [133, 138].

Manufacturing intelligence therefore cannot be interpreted as statically programmed behavior embedded within algorithms alone. Rather, it is continuously re‐parameterized through recursive interactions among computational abstraction, embodied execution, and evolving physical environments. This transition fundamentally changes the architecture of intelligent manufacturing systems, shifting them from sequential automation pipelines toward distributed adaptive ecosystems capable of continuous self‐modification [113, 116].

#### 6.2.2 Asymmetry Between AI, EI, and PI

Although AI, EI, and PI participate in a unified co‐evolutionary framework, the three domains are fundamentally asymmetric in both functionality and informational structure. This asymmetry is essential because each intelligence domain occupies a distinct position along the spectrum between abstraction and physical grounding [138].

AI primarily operates within high‐dimensional representation spaces characterized by strong abstraction capability but limited intrinsic physical grounding. Its strength lies in extracting latent correlations, constructing predictive models, and performing large‐scale optimization across complex manufacturing environments [95, 128, 130]. However, because computational reasoning is fundamentally separated from direct physical interaction, AI systems often struggle to maintain robustness under distribution shifts, environmental perturbations, and unmodeled physical dynamics [133, 159]. As a result, purely AI‐driven manufacturing architectures frequently exhibit brittle generalization when deployed in open and evolving industrial environments.

In contrast, PI resides at the opposite end of the abstraction spectrum. Physical intelligence emerges directly through material organization, structural mechanics, multiphysics coupling, and environmental interaction [116, 138]. Such systems possess exceptionally high physical grounding because intelligent behavior is intrinsically embedded within matter itself. Mechanical compliance, self‐regulation, adaptive deformation, and energy‐efficient response can therefore emerge without explicit centralized computation [117, 195]. Yet PI alone possesses limited abstraction capability and relatively low programmability, restricting its capacity for long‐horizon planning, symbolic reasoning, and generalized adaptation across heterogeneous manufacturing scenarios.

EI occupies an intermediate and fundamentally mediating role between these two extremes. Rather than functioning merely as an execution interface, EI serves as an adaptive translation layer that continuously compresses, reconstructs, and operationalizes information across digital and physical domains [137, 147]. Embodied systems translate abstract computational policies into physically realizable behaviors while simultaneously converting high‐dimensional physical interactions into compact and actionable representations for AI systems. Through this mediation process, EI enables the bidirectional transfer of information between symbolic reasoning and material response.

This asymmetry directly explains why isolated optimization of AI, EI, or PI alone is fundamentally insufficient for achieving fully adaptive manufacturing intelligence. AI‐only systems lack robust physical grounding, EI‐only systems remain constrained by limited cognitive horizons, and PI‐only systems suffer from insufficient programmability and abstraction capability. No single intelligence domain can simultaneously achieve scalable abstraction, adaptive embodiment, and physically realizable functionality under complex manufacturing conditions [133, 138].

Co‐evolution therefore becomes a theoretical necessity rather than a technological preference. Intelligent manufacturing systems can only achieve robust adaptation by continuously redistributing functionality across computational cognition, embodied interaction, and material intelligence. In this framework, intelligence is not localized within any single layer, but emerges from the dynamic coordination of asymmetrically coupled subsystems operating across multiple physical and informational scales [113, 116, 148].

#### 6.2.3 Multi‐Timescale Intelligence Dynamics

Beyond functional asymmetry, AI, EI, and PI also evolve on fundamentally different temporal scales. Intelligent manufacturing systems should therefore be interpreted not as static architectures, but as multi‐timescale dynamical systems in which computational learning, embodied adaptation, and material response evolve concurrently yet asynchronously [138].

AI primarily operates on relatively slow temporal scales associated with offline optimization, model updating, long‐horizon knowledge accumulation, and cross‐domain representation learning [130, 131, 132]. Computational intelligence typically requires iterative training, large‐scale data aggregation, and gradual refinement of predictive structures over extended operational periods. Consequently, AI governs the slow evolution of manufacturing cognition and strategic adaptation across production lifecycles.

EI functions on intermediate temporal scales associated with real‐time control, embodied interaction, adaptive execution, and closed‐loop behavioral coordination. Embodied systems must continuously respond to changing production conditions, sensory feedback, and environmental perturbations during active operation [137, 147]. Unlike AI, which often optimizes over extended temporal horizons, EI prioritizes rapid situational adaptation and online behavioral adjustment under physical constraints.

PI spans mechanism‐dependent temporal scales since its response is governed by the intrinsic dynamics of the relevant physical structure. Elastic instabilities may occur rapidly, whereas thermal diffusion can require longer times [116, 138, 195]. In physically intelligent systems, adaptation may therefore emerge directly from matter itself through localized interactions between material architecture and environmental stimuli [117, 122].

The coexistence of these heterogeneous temporal scales fundamentally alters the organization of manufacturing intelligence. Rather than relying on simultaneous centralized computation, intelligent behavior emerges through synchronization across slow computational learning, intermediate embodied adaptation, and fast physical response. Short‐timescale material adaptation can stabilize embodied interaction before higher‐level computational reasoning is updated, while long‐timescale AI optimization gradually reshapes future embodiment strategies and physical configurations [133, 136]. Manufacturing intelligence thus becomes temporally distributed across nested adaptive processes operating at different rates but remaining continuously coupled.

This perspective upgrades intelligent manufacturing from a static cyber–physical architecture into a continuously evolving dynamical ecosystem. Intelligence is no longer defined solely by instantaneous computational capability, but by the capacity of the overall system to coordinate learning, embodiment, and physical adaptation across heterogeneous temporal scales under real‐world uncertainty [113, 138].

### 6.3 Lifecycle Embedding of AI–EI–PI Co‐Evolution

The co‐evolution of artificial intelligence (AI), embodied intelligence (EI), and physical intelligence (PI) cannot be fully understood in isolation from the manufacturing lifecycle itself. In advanced manufacturing systems, intelligence does not emerge at a single operational stage, nor does it remain statically localized within computational models, robotic platforms, or material architectures. Instead, co‐evolution unfolds continuously across design, fabrication, operation, and maintenance, where each stage recursively reshapes the informational, embodied, and physical conditions governing subsequent stages [113, 135]. Manufacturing intelligence therefore becomes lifecycle‐embedded rather than stage‐specific, allowing cognition, interaction, and material adaptation to evolve together throughout the existence of the system (Figure 10).

**FIGURE 10:**
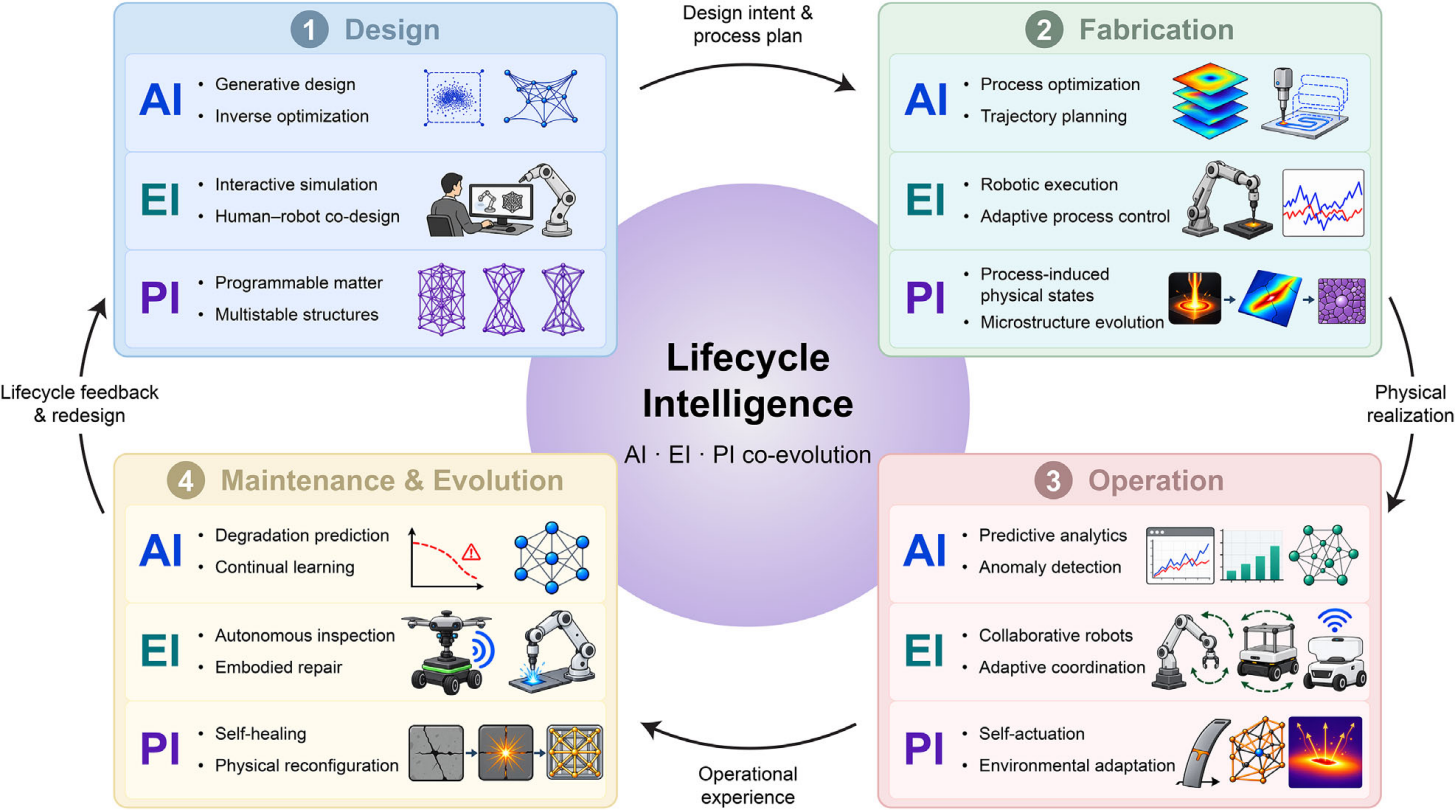
Recursive lifecycle embedding of AI–EI–PI co‐evolution in intelligent manufacturing ecosystems.

#### 6.3.1 Design Phase

The design phase represents the earliest stage at which AI–EI–PI co‐evolution begins to emerge. Conventional engineering design has historically focused on geometric optimization, where structures are synthesized primarily according to static performance metrics under predefined constraints. However, the integration of AI, EI, and PI fundamentally transforms design from geometry‐oriented optimization toward behavior‐oriented intelligence generation [95, 148].

Within this framework, AI contributes generative design, inverse optimization, surrogate modeling, and high‐dimensional exploration of multifunctional design spaces. Rather than merely identifying optimal geometries, AI increasingly predicts and synthesizes the coupled relationships among material composition, structural organization, fabrication pathways, and operational behavior [136, 165]. Design thus evolves from static parameter optimization toward the generation of adaptive physical functionality.

At the same time, EI introduces interactive simulation, embodied design exploration, and human–machine collaborative reasoning into the design process. Embodied systems enable designers and intelligent agents to iteratively evaluate physical feasibility, environmental interaction, and execution constraints through closed‐loop virtual and physical interaction [132, 137]. This interactional capability significantly reduces the separation between abstract computational optimization and physically realizable manufacturing strategies.

PI further expands the design space by embedding programmable physical behavior directly into material and structural architectures. Material‐aware geometries, compliant mechanisms, multistable structures, and environmentally responsive matter allow functionality to emerge intrinsically through physical organization rather than exclusively through external control [116, 117, 122]. Consequently, the objective of design is no longer limited to defining static forms, but increasingly focuses on encoding adaptive behavior, environmental responsiveness, and embodied functionality into the physical substrate itself.

The convergence of these three domains fundamentally changes the nature of manufacturing design. Intelligence is no longer externally imposed onto passive structures after fabrication. Instead, design increasingly becomes the process through which computational reasoning, embodied interaction, and physical adaptation are co‐generated from the outset [135, 138].

#### 6.3.2 Fabrication Phase

The fabrication phase represents a critical transition during which abstract manufacturing intelligence becomes physically instantiated through material transformation and embodied execution. In traditional manufacturing systems, fabrication is often interpreted as a downstream realization step that passively implements predefined design specifications. Under the AI–EI–PI framework, however, fabrication itself becomes an active intelligence‐generation process [135, 171].

AI contributes to this stage through process optimization, adaptive planning, parameter prediction, and real‐time decision support. Learning‐based models continuously infer optimal fabrication trajectories, thermal conditions, deposition strategies, and machine coordination policies under uncertain and dynamically evolving process environments [125, 145, 146]. As manufacturing complexity increases, AI increasingly operates not as a static controller, but as an adaptive cognitive engine capable of continuously updating fabrication strategies in response to process feedback.

EI operationalizes these computational strategies through robotic execution, adaptive process control, and embodied environmental interaction. Embodied manufacturing systems continuously sense, interpret, and respond to changing fabrication conditions during operation [137, 159]. Robotic manipulators, autonomous manufacturing agents, and cyber–physical systems therefore act not merely as execution interfaces, but as physically adaptive mediators that translate computational intent into real‐world manufacturing behavior [147].

Simultaneously, PI emerges through process‐dependent material evolution and structural encoding. During fabrication, material properties, residual stresses, thermal histories, microstructural organization, and geometric morphology evolve dynamically under process conditions [135, 171]. These physical transformations directly influence the future behavior and adaptive capabilities of the manufactured system. Fabrication therefore does not simply produce physical objects; it embeds intelligence into matter through the coupled evolution of process conditions, structural organization, and material response [116, 117].

This perspective fundamentally redefines fabrication as a co‐evolutionary process in which computation, embodiment, and physical matter continuously interact to shape the final functionality of the manufacturing system. Intelligence is thus generated not only before fabrication through design optimization, but also during fabrication through embodied adaptation and material evolution [138].

#### 6.3.3 Operation Phase

The operation phase represents the stage at which manufacturing intelligence becomes fully distributed throughout the active production environment. Once manufacturing systems are deployed, intelligent behavior no longer arises solely from centralized computational reasoning, but from continuous interaction among predictive cognition, embodied coordination, and adaptive physical response [113].

AI contributes to operational intelligence through predictive analytics, decision‐making, anomaly detection, and long‐horizon optimization. By continuously analyzing heterogeneous operational data streams, AI systems infer future system states, optimize resource allocation, and support adaptive production management under uncertain and evolving environmental conditions [131, 142, 182]. However, computational prediction alone is insufficient for maintaining robust operation in dynamic physical environments.

EI provides the embodied infrastructure required for closed‐loop interaction and autonomous coordination. Intelligent robotic systems, collaborative manufacturing agents, and cyber–physical platforms continuously adapt their behavior through real‐time interaction with machines, humans, and surrounding environments [137, 147]. Rather than executing static predefined commands, embodied systems dynamically negotiate uncertainty, compensate for environmental perturbations, and coordinate distributed operational behaviors across manufacturing networks.

PI further decentralizes intelligence by embedding adaptive response directly into physical matter and structural organization. Self‐actuating materials, compliant architectures, mechanically adaptive systems, and environmentally responsive structures enable manufacturing platforms to locally regulate behavior without relying exclusively on centralized computation [116, 138, 195]. In such systems, physical adaptation emerges directly through intrinsic material and structural dynamics, allowing rapid response to environmental changes at minimal computational cost.

As a result, operational intelligence becomes spatially distributed across algorithms, embodied agents, and physical substrates simultaneously. Manufacturing systems increasingly behave as adaptive ecosystems in which sensing, decision‐making, actuation, and physical response are continuously integrated across multiple organizational layers [113, 138].

#### 6.3.4 Maintenance and Evolution Phase

The maintenance and evolution phase extends manufacturing intelligence beyond static operational stability toward continuous system adaptation and self‐evolution. Conventional maintenance strategies are primarily reactive, relying on periodic inspection and corrective intervention after degradation has already occurred. In contrast, AI–EI–PI co‐evolution enables manufacturing systems to continuously monitor, adapt, repair, and reconfigure themselves throughout extended operational lifecycles [131, 142, 183].

AI supports this transition through degradation prediction, continual learning, anomaly forecasting, and adaptive knowledge updating. By integrating historical operational data with real‐time monitoring information, AI systems progressively refine their understanding of failure mechanisms, material aging, and environmental influence [182, 184]. Maintenance therefore evolves from static fault detection toward predictive and self‐improving lifecycle management.

EI contributes through adaptive inspection, autonomous diagnosis, and embodied repair capabilities. Intelligent robotic agents can perform distributed inspection, interact with damaged environments, and execute localized repair strategies under uncertain operational conditions [137, 147]. Because embodied systems maintain continuous physical interaction with manufacturing environments, they provide the adaptive interface required for translating predictive knowledge into real‐world maintenance actions.

PI further enables physically adaptive recovery through self‐healing materials, reconfigurable architectures, and intrinsically adaptive structural responses. Material systems capable of autonomous crack closure, stress redistribution, thermal recovery, or environmentally triggered reconfiguration reduce dependence on external intervention and enhance long‐term operational resilience [116, 122, 138]. In such systems, recovery mechanisms are partially embedded within matter itself rather than being imposed entirely through external control.

Consequently, manufacturing systems begin to evolve from statically maintained infrastructures toward continuously adaptive and self‐evolving ecosystems. Intelligence no longer terminates once fabrication and deployment are completed. Instead, learning, embodiment, and material adaptation persist throughout the operational lifetime of the system, enabling manufacturing platforms to progressively reorganize their own functionality in response to changing environments, accumulated experience, and evolving physical conditions [113, 138].

### 6.4 Emergent System‐Level Properties From Co‐Evolution

The integration of AI, EI, and PI does not merely improve individual manufacturing functions; rather, it gives rise to emergent system‐level capabilities that cannot be achieved through isolated optimization of computational algorithms, embodied machines, or programmable materials alone [138]. Within co‐evolving manufacturing ecosystems, intelligence increasingly manifests as a distributed property arising from the continuous interaction between digital representation, physical embodiment, and material adaptation. As a result, system performance becomes governed not only by computational accuracy or mechanical precision, but also by the collective capacity of the entire manufacturing architecture to reorganize itself under uncertainty, environmental perturbation, and evolving operational demands [116, 148].

One of the most significant emergent properties is robustness. Conventional manufacturing systems often rely on rigid pre‐defined workflows that exhibit limited tolerance to unexpected disturbances, model mismatch, or environmental variability. In contrast, AI–EI–PI co‐evolution enables resilience through distributed adaptation across multiple layers simultaneously [128]. AI systems continuously update predictive models and decision boundaries based on real‐time feedback, EI systems dynamically modify control strategies and interaction behaviors, while PI systems physically absorb or redistribute disturbances through adaptive material responses and morphology‐dependent compliance [116, 147, 159]. Robustness therefore no longer emerges solely from redundancy or conservative design margins, but from the capacity of the entire system to continuously reorganize its internal representation, embodiment, and physical configuration in response to changing conditions.

Co‐evolution also fundamentally enhances adaptability. Traditional automation architectures typically operate within narrowly defined task domains because intelligence remains tightly coupled to fixed sensing modalities, predetermined geometries, and static process assumptions. By contrast, the integration of computational learning, embodied interaction, and programmable matter enables manufacturing systems to adapt across tasks, environments, and physical configurations [117, 130, 137]. AI‐driven representation learning allows knowledge transfer across heterogeneous operational scenarios, EI enables context‐aware behavioral adaptation during interaction and execution, and PI introduces morphology‐dependent functionality that alters system behavior directly at the material level. Importantly, adaptation is no longer restricted to software reconfiguration alone; instead, it becomes physically grounded through the dynamic interplay between geometry, material response, and embodied control. Such morphology‐aware adaptability allows manufacturing systems to exploit physical intelligence embedded within structures and materials rather than relying exclusively on centralized computation [136, 138].

Another emergent capability is the improvement of system‐level efficiency. In conventional manufacturing architectures, increasing intelligence often requires additional sensing, higher computational overhead, and increasingly centralized control infrastructures. However, co‐evolving systems partially redistribute computation and decision‐making into physical and embodied domains [116, 138]. Programmable materials can directly encode environmental responsiveness, thereby reducing the sensing and control burden imposed on external algorithms. EI further enables local adaptation through embodied interaction, allowing robotic systems to exploit mechanical compliance, passive dynamics, and environmental coupling to simplify control complexity [137, 195]. Meanwhile, AI coordinates global optimization and long‐horizon planning without requiring exhaustive centralized supervision of every low‐level physical process [132]. This redistribution of intelligence across computational, embodied, and material layers substantially reduces energetic, informational, and computational costs while simultaneously improving scalability and operational flexibility.

Perhaps the most transformative emergent property is autonomy. In layered automation systems, autonomy is typically interpreted as the ability to execute pre‐programmed tasks without human intervention. Under the AI–EI–PI co‐evolution framework, autonomy instead becomes a continuous process of self‐optimization, self‐reconfiguration, and system‐level evolution [147]. AI enables continual learning and adaptive decision‐making, EI facilitates autonomous coordination and closed‐loop interaction with complex environments, and PI introduces physically adaptive functionalities such as self‐actuation, self‐regulation, and self‐healing [122, 131, 137]. The resulting manufacturing ecosystem is therefore not statically maintained, but continuously evolves through recursive feedback between computation, embodiment, and material response. Intelligence becomes intrinsically embedded within the operational dynamics of the system itself rather than externally imposed through centralized control architectures [138].

Collectively, these emergent properties indicate a broader transition in the conceptual foundations of intelligent manufacturing. The ultimate objective is no longer merely the automation of production processes, but the development of manufacturing ecosystems capable of continuously learning, adapting, and physically evolving across scales [129]. Within such systems, intelligence is distributed across algorithms, machines, materials, and environments, enabling manufacturing architectures that are not only autonomous and efficient, but also resilient, adaptive, and evolutionarily responsive to the complexities of real‐world operation [116, 138].

### 6.5 Unified Architecture and Future Outlook

The AI–EI–PI framework proposed in this Review establishes a unified perspective for understanding manufacturing intelligence as a continuously evolving and physically grounded ecosystem rather than a collection of isolated technologies. Instead of interpreting artificial intelligence, embodied intelligence, and physical intelligence as sequential functional modules arranged within a hierarchical automation pipeline, the present framework positions them as dynamically coupled domains whose interactions collectively determine the adaptability, robustness, and autonomy of manufacturing systems [113, 138]. Intelligence therefore emerges not from any single computational model, robotic platform, or programmable material, but from the recursive exchange of information, constraints, and physical feedback across all layers of the manufacturing architecture.

In this framework, AI operates primarily within the representation and abstraction domain, enabling long‐horizon learning, predictive reasoning, and generative optimization [95, 130, 132]. EI functions as the embodied mediation layer that translates computational representations into adaptive physical interaction through sensing, control, and autonomous execution [137, 147]. PI provides the material realization layer in which physical response, structural adaptation, and environmental coupling emerge directly through programmable matter and morphology‐dependent behavior [116, 117, 138]. Crucially, the framework emphasizes that information flow is not unidirectional. Instead, continuous feedback between AI, EI, and PI reshapes the operational space of the entire manufacturing system throughout design, fabrication, operation, maintenance, and long‐term evolution.

The proposed architecture further highlights the importance of distributed intelligence across heterogeneous temporal and physical scales. AI‐driven learning and optimization typically evolve over relatively slow timescales associated with data accumulation and model updating, whereas EI enables intermediate‐timescale adaptation through real‐time interaction and control. In contrast, PI operates at fast physical timescales governed by material deformation, thermal response, mechanical compliance, and environmental coupling [133, 138]. Manufacturing intelligence therefore emerges through synchronization across these heterogeneous dynamical processes rather than through centralized computation alone. This temporal hierarchy fundamentally distinguishes co‐evolving manufacturing ecosystems from conventional automation architectures, where intelligence is often localized within static software infrastructures detached from material and environmental dynamics [98, 128].

Another important implication of the unified framework is the transition from externally imposed intelligence toward intrinsically embedded intelligence. In traditional intelligent manufacturing systems, algorithms are typically deployed as supervisory layers operating above passive machines and materials. Such architectures inherently separate cognition from physical realization, thereby limiting adaptability under uncertainty and constraining the scalability of autonomous operation [159]. By contrast, the AI–EI–PI paradigm distributes intelligence directly across algorithms, robotic embodiment, structural morphology, and programmable materials [116, 117, 137]. Physical systems no longer merely execute computational commands; they actively participate in sensing, adaptation, decision‐making, and information generation. As a result, manufacturing systems increasingly resemble adaptive ecological networks in which computational cognition, embodied interaction, and material evolution continuously co‐organize across scales.

The convergence of AI, EI, and PI also opens new opportunities for fundamentally rethinking the objectives of advanced manufacturing. Future systems may no longer be optimized solely for precision, throughput, or automation efficiency, but for long‐term adaptability, evolutionary resilience, and autonomous self‐reconfiguration under dynamically changing operational environments [132]. Such systems could continuously modify their geometry, material composition, control policy, and functional behavior in response to environmental perturbations, task variation, and accumulated operational experience [116, 122, 136]. In this context, manufacturing becomes not only a process of producing intelligent artifacts, but also a process through which intelligence itself continuously evolves through recursive interaction between digital models, embodied agents, and adaptive matter.

Ultimately, future manufacturing systems will not be defined solely by increasingly powerful algorithms or increasingly automated machines, but by the co‐evolution of computational cognition, embodied interaction, and physically adaptive matter. Within this emerging paradigm, intelligence is no longer externally imposed onto manufacturing infrastructures as an additional computational layer. Instead, it becomes intrinsically embedded throughout algorithms, machines, materials, and processes across the entire manufacturing lifecycle, enabling the emergence of manufacturing ecosystems capable of continuous learning, adaptation, and physical evolution across scales [113, 138].

## 7 Challenges and Future Directions

Although the convergence of artificial intelligence (AI), embodied intelligence (EI), and physical intelligence (PI) opens a compelling route toward next‐generation manufacturing, they are still weakly integrated in manufacturing practice. The central challenge is not simply to make manufacturing systems more automated or more data‐rich, but to determine how intelligence can be rigorously distributed across models, machines, materials, and environments in ways that are physically grounded, operationally reliable, and scientifically interpretable. Advancing this vision will require progress on several fronts, spanning theory, system architecture, materials design, and industrial deployment. Each subsection below is stated as technical bottlenecks or industrial thresholds. The AI–EI–PI framing is used to separate gaps that remain inside computational AI from gaps that appear only at the interfaces among models, embodied loops, and physical substrates.

### 7.1 Toward a Rigorous Science of Manufacturing Intelligence

A first unresolved issue is conceptual as much as technical: there is still no unified scientific language for describing intelligence across computational, embodied, and material substrates. In current practice, “intelligence” may refer to prediction accuracy, adaptive control, autonomous decision‐making, self‐regulation, or even stimulus‐responsive material behavior, depending on disciplinary context. This ambiguity limits comparison across systems and obscures what is genuinely new when intelligence shifts from software to morphology and ultimately to matter. Without operational criteria, a vision model, a compliant robot, a digital twin, and a self‐healing polymer can all be counted as evidence of manufacturing intelligence, which prevents any ranking of industrial readiness.

Future work should move toward a rigorous theory of manufacturing intelligence that explicitly characterizes where information is stored, how it is transformed, and through which physical channels it influences behavior. Such a framework should distinguish between symbolic inference, sensorimotor adaptation, and intrinsic material response while also clarifying how these layers interact over time. This would allow manufacturing systems to be evaluated not only by conventional metrics such as throughput, precision, and defect rate, but also by higher‐order properties including physical adaptability, self‐regulation capacity, response latency, energetic efficiency, and resilience under uncertainty. In this sense, the next step for the field is not only better tools, but a more precise scientific account of what it means for a manufacturing system to be intelligent. A near‐term task is therefore to report, for each claimed function, where the mapping from observation to action is computed, whether the body–environment loop is closed online, and whether any part of that mapping is physically instantiated.

### 7.2 Bridging Scales from Data to Matter

A second major challenge is that AI, EI, and PI couple across scales, and the interfaces are rarely measured. AI, EI, and PI operate over fundamentally different spatial and temporal regimes: AI often acts on high‐dimensional data and abstract latent representations; EI unfolds through robot bodies, sensors, and machine‐environment interaction; PI emerges from local mechanics, transport, chemistry, and structural response. Integrating these forms of intelligence requires more than interface engineering. It requires a cross‐scale language capable of linking abstract design intent to embodied behavior and ultimately to material transformation.

This is particularly important in advanced manufacturing, where small changes in composition, microstructure, interface condition, or thermal history can lead to large changes in macroscopic performance. Current systems often treat these scales separately, with design optimization, process planning, machine control, and material response handled in largely disconnected pipelines. Future manufacturing platforms should instead be able to propagate constraints and opportunities bidirectionally across scales: material states should inform control; process history should inform structure; structural response should inform design generation. Achieving this level of integration will likely depend on hybrid frameworks that combine mechanistic models, in‐situ sensing, differentiable simulation, and adaptive generative design into a single physically coherent loop. The missing measurements are interface‐specific: the delay with which contact updates a learned policy (AI–EI); the characteristic time of snap‐through, heat transfer, or healing relative to controller bandwidth (EI–PI); and whether a generated morphing or recovery function survives fabrication (AI–PI). Until these quantities are reported, claims of cross‐scale integration remain architectural rather than experimental.

### 7.3 From Data Dependence to Physics‐Grounded Generalization

One of the most persistent bottlenecks in intelligent manufacturing remains generalization. Models trained under one machine configuration, material system, or environmental condition often degrade sharply when transferred to another. This is not simply a machine learning problem; it reflects a deeper mismatch between statistical pattern extraction and the causal physical structure of manufacturing processes.

A key direction for the field is therefore to reduce dependence on exhaustive data collection by increasing the amount of intelligence that is physically constrained from the outset. This includes embedding conservation laws, constitutive relations, process windows, and stability criteria directly into design, monitoring, and control frameworks. Physics‐informed learning can reduce data demand, but incomplete models and process‐specific calibration can leave a physically regularized predictor as brittle as a statistical one after a machine or batch change. The useful distinction is among three remedies that are often conflated: better priors and transfer within AI; online correction through embodied interaction; and delegation of selected local responses to matter. None of these automatically supplies the others.

### 7.4 Designing Intelligence Into Materials and Structures

If PI is to become more than an evocative concept, the field must learn how to engineer intelligence directly into material and structural form with the same rigor currently applied to algorithm and machine design. At present, many physically intelligent systems remain proof‐of‐concept demonstrations: shape‐changing architectures, self‐healing materials, multistable mechanisms, and responsive metamaterials have shown remarkable functionality, but they are often difficult to scale, difficult to manufacture reproducibly, and difficult to integrate into industrial workflows.

A near‐term bottleneck is the development of manufacturing‐ready physically intelligent matter. This includes materials that can sense load, store mechanical history, adapt stiffness, localize damage, recover function, or reconfigure geometry without requiring dense external instrumentation or centralized control. More importantly, these capabilities must be designed not as isolated features but as integrated physical programs. In future systems, geometry, composition, interface architecture, and fabrication pathway may be co‐designed to encode response rules directly into matter. Manufacturing would then move beyond producing intelligent devices toward producing materials and structures that are themselves capable of situated adaptation. The nearer adoption barriers are more specific: fatigue under cyclic loads, scatter in activation thresholds, limited reversibility, poor reprogrammability, and the absence of acceptance tests for a physically encoded function after fabrication. If such an element is placed in a robot or fixture, the controller must also know its timescale, force range, and failure mode; otherwise PI enters the loop as an unexplained disturbance.

### 7.5 Manufacturing Systems That Learn Through Action

A further limitation of many current approaches is that learning is still treated as something that occurs primarily before deployment, during offline training or virtual optimization. Manufacturing, by contrast, is inherently interactive: systems encounter drift, disturbance, wear, contamination, variability, and unforeseen coupling effects throughout their operational life. Truly adaptive manufacturing will therefore require systems that learn not only from datasets, but from action itself.

Even though closed‐loop sensing and actuation are already standard in manufacturing, an open issue remains that learned policies, operator judgment, and material states are still weakly coupled to those existing loops. The technical gap is not the absence of feedback, but the constraints on using it: exploration can scrap parts or damage tools, and wear or a responsive insert can change the plant seen by the learner. Near‐term work is therefore a bounded identification and safety problem—which loop produced a given correction, and whether that update remains valid after contact, drift, or material change—rather than a general vision of factories that improve by being used.

### 7.6 Human‐Centered Intelligence Beyond Supervision

Although much discussion of manufacturing autonomy focuses on reducing human intervention, the long‐term trajectory of Industry 5.0 is unlikely to be defined by human removal alone. The more consequential question is how human judgment, tacit knowledge, creativity, and ethical responsibility can be coupled productively with machine and material intelligence.

Future manufacturing systems should therefore be designed not only for autonomous function but for meaningful human participation. This will require new interfaces through which operators can understand, interrogate, and shape the behavior of intelligent systems across digital, embodied, and physical layers. In particular, an operator cannot usefully supervise a distributed system if it is unclear whether an action came from a model update, an embodied policy, or a material response. It will also require systems capable of adapting to human variability rather than forcing humans to adapt to machine rigidity. In the long run, the most effective manufacturing ecosystems may not be those that eliminate the human from the loop, but those that place human expertise within a richer ecology of computational, embodied, and material agency.

### 7.7 Trust, Verification, and Industrial Translation

For all its promise, intelligent manufacturing will not be adopted at scale unless it can be trusted under real industrial conditions. This remains a substantial challenge, particularly as intelligence becomes distributed across black‐box models, embodied hardware, and responsive materials. Failures in such systems may not arise from a single identifiable source, but from interactions among learned policies, physical dynamics, fabrication variability, and environmental perturbations.

Future progress will therefore depend on new approaches to verification and validation that extend beyond software benchmarking. Manufacturing intelligence must be tested not only for performance under nominal conditions, but for stability, recoverability, and graceful degradation under off‐nominal scenarios. This is especially important for safety‐critical sectors such as aerospace, biomedical manufacturing, infrastructure, and energy systems. What is missing is layer‐crossing evidence: uncertainty that travels from the model into the controller, recoverability after contact or material fatigue, and acceptance tests for a physically encoded function after fabrication, not only for the as‐designed geometry. These are needed if AI–EI–PI systems are to move from laboratory prototypes to industrial infrastructure.

### 7.8 Toward Self‐Evolving Manufacturing Ecosystems

Looking forward, the most ambitious frontier is not a smarter machine, a better robot, or a more responsive material in isolation. It is the emergence of manufacturing ecosystems in which intelligence is distributed across the full stack of design, matter, machine, and operation. In such systems, design models would generate not only shapes but adaptive physical functions; embodied platforms would not merely execute instructions but negotiate with uncertainty in real time; and materials would not remain passive substrates but participate directly in sensing, memory, regulation, and recovery.

This points toward a future of self‐evolving manufacturing ecosystems: factories that do not simply produce objects, but continuously reorganize their own methods of production; materials that are fabricated with latent adaptive capability; and products whose intelligence begins not after manufacturing, but during manufacturing itself. At that point, manufacturing will no longer be understood solely as the transformation of matter into form. It will become the process by which matter, machine, and model are jointly organized into systems capable of adaptation, interaction, and purposeful physical behavior.

In this view, the ultimate opportunity of AI–EI–PI convergence is not only to improve manufacturing efficiency, but to redefine manufacturing as a generative science of intelligence in the physical world. Self‐evolution is nevertheless a long‐horizon program rather than a near‐term objective. The blocking issues remain the interface and adoption gaps above: models that do not transfer, embodied loops that cannot explore safely, materials that cannot be qualified, and failures that cannot be assigned to a layer. Until those problems are reduced, the actionable question is which functions can already be redistributed from centralized computation to embodied interaction or to matter without loss of traceability.

## 8 Conclusion

This review treats manufacturing intelligence as three implementation modes: artificial intelligence (AI), embodied intelligence (EI), and physical intelligence (PI). In this context, artificial intelligence, embodied intelligence, and physical intelligence should not be viewed as competing directions, but as complementary forms of capability that operate across different layers of manufacturing reality.

This review has argued that the future of manufacturing intelligence lies in the integration of these three domains. Artificial intelligence provides abstraction, prediction, and generative reasoning at scale. Embodied intelligence enables those capabilities to be enacted in dynamic physical environments through sensing, action, and interaction. Physical intelligence extends this logic deeper into matter, where response, adaptation, and regulation can arise directly from material and structural design.

The significance of this shift is not only technological, but conceptual. It changes how manufacturing systems are designed, how autonomy is achieved, and where adaptation is expected to occur. Instead of treating physical systems as passive recipients of optimized commands, next‐generation manufacturing will increasingly rely on systems that can sense through form, compute through interaction, and respond through physics. Such systems are likely to be more robust to uncertainty, less dependent on exhaustive data and control, and better aligned with the demands of resilient, sustainable, and human‐centered production.

Realizing this vision will require closer integration across fields that have often developed separately, including artificial intelligence, robotics, mechanics, materials science, additive manufacturing, and cyber–physical systems. It will also require new methods for co‐design, where algorithms, machines, materials, and fabrication pathways are developed not sequentially, but as interdependent components of a single intelligent system. The key challenge ahead is therefore not simply to build smarter models, but to engineer smarter manufacturing matter and smarter manufacturing bodies.

The long‐term trajectory is clear: manufacturing intelligence is moving from being externally imposed to being physically realized. The most capable manufacturing systems of the future may not be those that compute the most, but those that most effectively unite digital cognition, embodied action, and material response into a coherent whole. In that sense, the next frontier of smart manufacturing is not only artificial intelligence in industry, but intelligence that is designed into the very fabric of industrial systems.

## Conflicts of Interest

The authors declare no conflicts of interest.

## Data Availability

All data supporting the findings of this study are included within the article.
